# C-Type Lectins Link Immunological and Reproductive Processes in *Aedes aegypti*

**DOI:** 10.1016/j.isci.2020.101486

**Published:** 2020-08-21

**Authors:** Hsing-Han Li, Yu Cai, Jian-Chiuan Li, Matthew P. Su, Wei-Liang Liu, Lie Cheng, Shu-Jen Chou, Guann-Yi Yu, Horng-Dar Wang, Chun-Hong Chen

**Affiliations:** 1 Institution of Biotechnology, National Tsing Hua University, Hsinchu, 300044, Taiwan; 2 National Institute of Infectious Diseases and Vaccinology, National Health Research Institutes, Miaoli 350401, Taiwan; 3 Temasek Life Sciences Laboratory, National University of Singapore, 117604, Singapore; 4 Department of Biological Sciences, National University of Singapore, 117558, Singapore; 5 Department of Biological Science, Nagoya University, Nagoya 464-8602, Japan; 6 National Mosquito-Borne Diseases Control Research Center, National Health Research Institutes, Miaoli 350401, Taiwan; 7 Institute of Plant and Microbial Biology, Academia Sinica, Taipei 115201, Taiwan

**Keywords:** Biological Sciences, Immunology, Microbiology Parasite

## Abstract

Physiological trade-offs between mosquito immune response and reproductive capability can arise due to insufficient resource availability. C-type lectin family members may be involved in these processes. We established a *GCTL-3*^*−/−*^ mutant *Aedes aegypti* using CRISPR/Cas9 to investigate the role of *GCTL-3* in balancing the costs associated with immune responses to arboviral infection and reproduction. *GCTL-3*^*−/−*^ mutants showed significantly reduced DENV-2 infection rate and gut commensal microbiota populations, as well as upregulated JAK/STAT, IMD, Toll, and AMPs immunological pathways. Mutants also had significantly shorter lifespans than controls and laid fewer eggs due to defective germ line development. dsRNA knock-down of *Attacin* and *Gambicin*, two targets of the AMPs pathway, partially rescued this reduction in reproductive capabilities. Upregulation of immune response following *GCTL-3* knock-out therefore comes at a cost to reproductive fitness. Knock-out of other lectins may further improve our knowledge of the molecular and genetic mechanisms underlying reproduction-immunity trade-offs in mosquitoes.

## Highlights

• mos*GCTL-3* mutants showed a reduced DENV-2 infection rate
• mos*GCTL-3* mutants had upregulated JAK/STAT, IMD, Toll, and AMPs pathway components
• mos*GCTL-3* knock-out led to reduced gut microbiota population sizes, and diversity
• mos*GCTL-3* regulates germ line development and influences fertility

## Graphical Abstract

**Figure:**
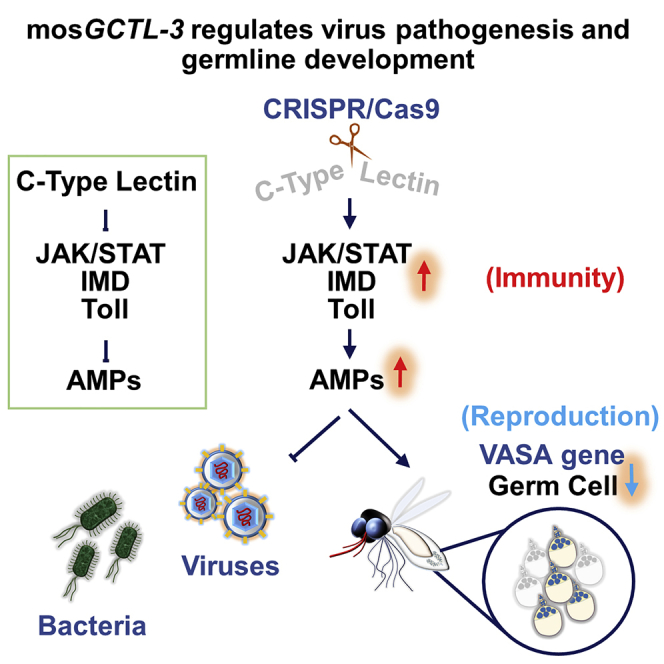

## Introduction

Physiological trade-offs between immunological response to infection and reproductive ability are likely the result of limited availability of energetic resources (Schwenke et al., 2016). Increased investment in the immune system should therefore result in decreased reproductive capabilities, and vice versa, although there are many other factors that influence the balance of resource allocation (including age and pathogen virulence). Understanding these trade-offs is essential for improving our knowledge of disease-transmitting mosquito species, which are constantly exposed to pathogens during blood feeding and whose egg-laying capabilities are highly relevant in terms of vector control (Delhaye et al., 2016; Flatt and Kawecki, 2007; Miyashita et al., 2019; Simmons, 2011).

Recent publications have highlighted the importance of the mosquito as a site of viral replication and have described methodologies that can inhibit or enhance virus replication within the mosquito itself (Buchman et al., 2019; Wang et al., 2017; Yen et al., 2018). These strategies affect a diverse range of targets but have often resulted in changes to mosquito reproductive potential via unknown mechanisms. Indeed, despite their importance, the wider mechanisms that underlie reproductive/immunological trade-offs remain largely unknown in mosquitoes (Hurd, 2002; Schwenke et al., 2016).

One pathway reported to heavily influence the immune response to infection involves C-type lectins (CTLs), a family of proteins that exhibit carbohydrate-binding activity and have been shown to play vital roles in immune activation and viral pathogenesis (Dambuza and Brown, 2015; Liu et al., 2014; Watanabe et al., 2006). At least 52 C-type lectin domain-containing proteins (CTLDcps) have been annotated in mosquitoes; these have been further categorized as CTLD-S, CTLD-E, CTLD-SP, and CTLD-X. CTLDcps expression levels can vary significantly across developmental stages (Adelman and Myles, 2018). CTLDcps have been identified as important for West Nile virus (WNV) replication and dengue virus (DENV) infection (Adelman and Myles, 2018). The functions of many CTLs remain unclear, however, particularly with regards to Zika virus (ZIKV) infection (Fontes-Garfias et al., 2017; Sirohi and Kuhn, 2017).

Many CTLs are employed as receptors or attachment factors to facilitate flavivirus invasion during infection. In previous studies, mosquito *GCTL-1* (mos*GCTL-1*) was shown to be recruited by mosquito protein tyrosine phosphatase-1 (mos*PTP-1*) to allow viral attachment of WNV to cells and facilitate viral entry (Cheng et al., 2010). Mosquito *GCTL-7* (mos*GCTL-7*) has also been reported to bind to the N154 site of N-glycan on the Japanese encephalitis virus envelope protein to promote viral entry into mosquitoes (Liu et al., 2017). Furthermore, two CTLD-S proteins, AAEL0011453 and AAEL012353, are thought to play a key role in gut microbiota homoeostasis and viral entry (Pang et al., 2016). Mosquito *GCTL-3* (mos*GCTL-3*, AAEL000535/AAEL029058), which belongs to the CTLD-S group, can bind to the envelope protein of DENV and assist in the viral infection of host cells. Treating *Aedes* mosquitoes with mos*GCTL-3* antisera was found to be sufficient to block DENV infection (Liu et al., 2014).

Mosquito CTLs also play an important role in maintaining gut microbiome homeostasis, with the microbiome heavily influencing viral replication. In particular, the mosquito gut commensal bacterium, *Serratia marcescens*, secretes the protein *Sm*Enhancin to facilitate arbovirus infection (Wu et al., 2019). *S. marcescens* has also been shown to cause disease in hosts and affect the growth, survival, and development of mosquito larvae (Patil et al., 2011). An abundance of other bacterial genera have additionally been detected in mosquito whole bodies, including *Shigella*, *Asaia,* and *Listeria* (Bertani, 2004; Wasilauskas et al., 1974).

mos*GCTL*s act as immune antagonists that can be utilized by the gut microbiome to escape the bactericidal ability of antimicrobial peptides (AMPs) to protect microbial flora (Pang et al., 2016; Zhang et al., 2017). AMPs expression levels, mediated via the JAK/STAT and Toll pathways, are significantly upregulated in DENV-infected mosquitoes, although DENV-infected cells also decrease the production of AMPs that are mediated via the IMD pathway (Anglero-Rodriguez et al., 2017; Kingsolver et al., 2013; Liu et al., 2012; Xiao et al., 2014; Zhang et al., 2017). The interactions between the different signaling pathways are highly complex and interrelated; further investigation of the influence of CTL family members on the mosquito immune system and gut microbiome composition, as well as the resulting effects on infection rate and transmission, could improve our understanding of these interactions.

We therefore used CRISPR/Cas9 to generate a mos*GCTL-3* knock-out mutant line in *Aedes aegypti*, a major vector of both dengue and ZIKVs (Anglero-Rodriguez et al., 2017; Guzman and Isturiz, 2010; Johansson et al., 2016), with the aim of investigating the trade-offs between immune response and reproduction. mos*GCTL-3* mutants showed a reduction both in DENV-2 and ZIKV prevalence of infection after a blood meal. Mutants also showed elevated JAK/STAT signaling and increased production of specific AMPs, as well as a reduction in gut microbiota, which potentially explains the reduction in DENV-2 prevalence of infection. However, mos*GCTL-3* mutants exhibited compromised germ line development and reduced fertility and were short-lived. Mutant reproductive capabilities were partially restored following dsRNA mediated knock-down of *Attacin* and *Gambicin*, downstream effectors of the AMPs pathway. Production of other CTL knock-out mosquito lines could provide more detail on the functions and mechanisms of this protein family and the role they play in balancing competition for resources between immune response and reproduction.

## Results

### Generation of *Aedes aegypti GCTL-3* Mutants by CRISPR/Cas9

**Figure 1:**
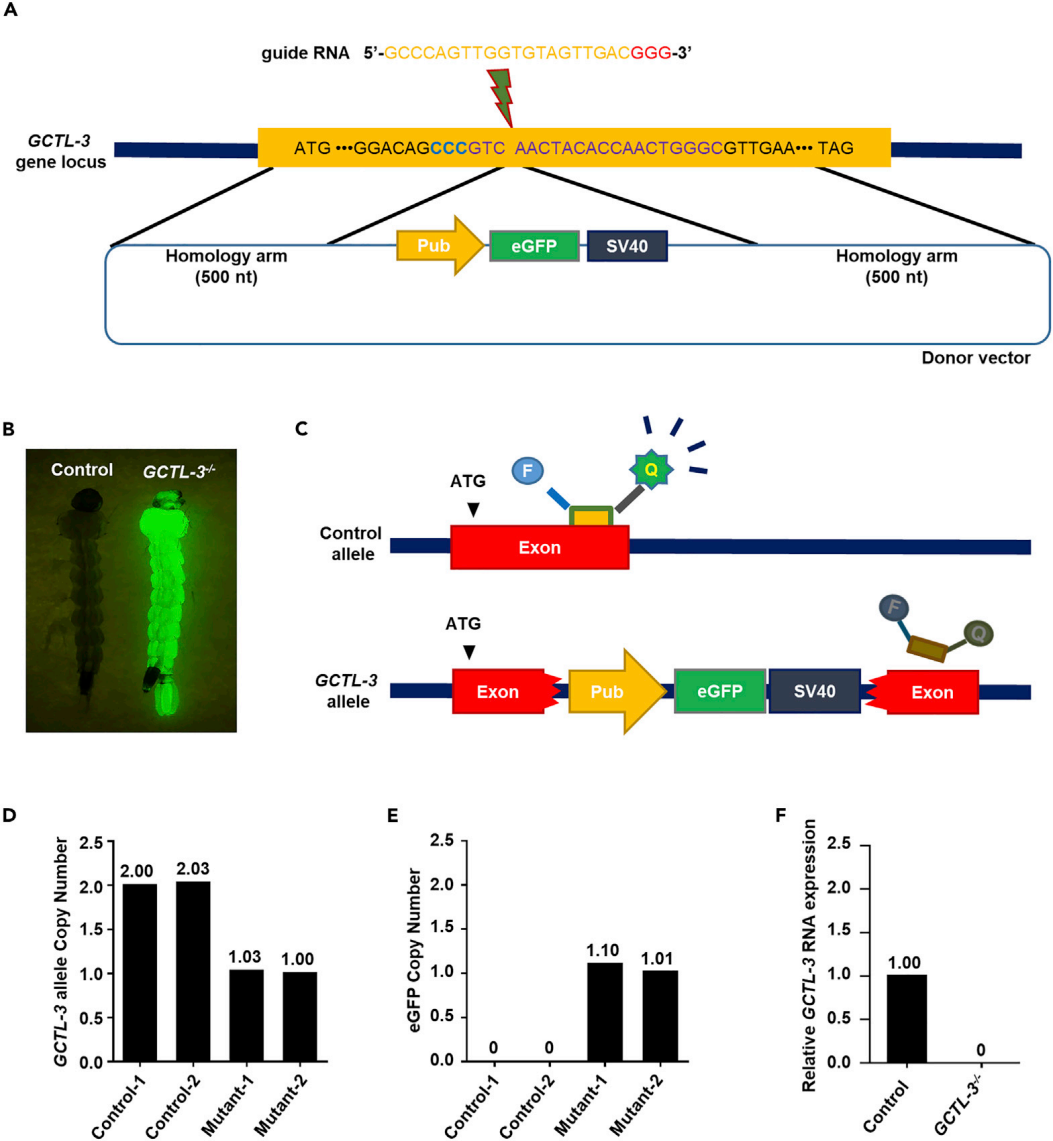
Generation of *Aedes aegypti GCTL-3* Knock-out by CRISPR/Cas9 (A) Schematic of the *A. aegypti GCTL-3* gene locus showing the sgRNA target site (red arrow). Homology arms correspond to sequences immediately adjacent to the predicted cut sites. (B) Generation of *A. aegypti GCTL-3* gene knock-out mutant mosquitoes: control larvae without fluorescence (left panel); expression of *eGFP* fluorescence in the whole bodies of mutant larvae driven by a poly-ubiquitin (PUb) promoter (right panel). (C) Schematic of allele-specific detection using TaqMan probes. The designed probe and primer sets for *eGFP* and *GCTL-3* are included in Supplemental Information, Table S3. (D and E) Copy number variants of (D) mos*GCTL-3* and (E) *eGFP* in control and heterozygote mutant mosquitoes (N=3); data are represented as mean ± SD. (F) mRNA expression levels of *GCTL-3* in control and mutant mosquitoes (N = 5 each) detected by qPCR across three biological replicates; data are represented as mean ± SD. See also Tables S1–S3.

Mutant generation in many model organisms commonly relies on combining single guide RNA (sgRNA)-mediated deletion with homologous recombination using a donor plasmid containing a selective marker (Supplemental Information, Table S1). Using a similar strategy, we here generated two *GCTL-3* mutants by inserting a cascade containing an *eGFP* gene under the control of a mosquito polyubiquitin promoter into the *GCTL-3* exon region (Figures 1A and 1B, Supplemental Information, Table S2).

To verify the deletion of *GCTL-3* in these mutants, as well as to check for potential off-target effects, we utilized a digital droplet PCR platform to determine the *eGFP* copy number (Figure 1C, Supplemental Information, Table S3). Both heterozygous mutant (*GCTL-3*^*+/−*^) mosquitoes had a single copy of *GCTL-3* and *eGFP* (Figures 1D and 1E), whereas control mosquitoes had two copies of *GCTL-3* (Figure 1D). We further used genomic PCR and sequencing to confirm that the five potential sgRNA target sites that contained similar sequences to *GCTL-3* were all intact in these two mutants (Supplemental Information, Figure S1A). We also confirmed the recombination site in *GCTL-3* knock-out mutant mosquitoes via PCR and sequencing (Supplemental Information, Figures S1B–S1F, Table S4). To investigate the function of *GCTL-3*, we selected one line (mutant-1) and performed outcrossing for five generations to establish the *GCTL-3*^*−/−*^ homozygous mutant line, and used it throughout this study (Supplemental Information, Figure S2). Homozygous mutant exhibited *eGFP* fluorescence throughout the whole body and did not express detectable *GCTL-3* transcripts (Figure 1F). We then tested heterozygous mosquitoes for fitness and reproductive phenotyping. We found no significant differences between wild-type controls and heterozygous mosquitoes, indicating that the possibility of a dominant phenotype due to the pub-EGFP marker was negligible (Supplemental Information, Figure S3 and Data S6).

### *GCTL-3*^*−/−*^ Mosquitoes Exhibited a Reduced Infection Rate for DENV, but Not ZIKV

**Figure 2:**
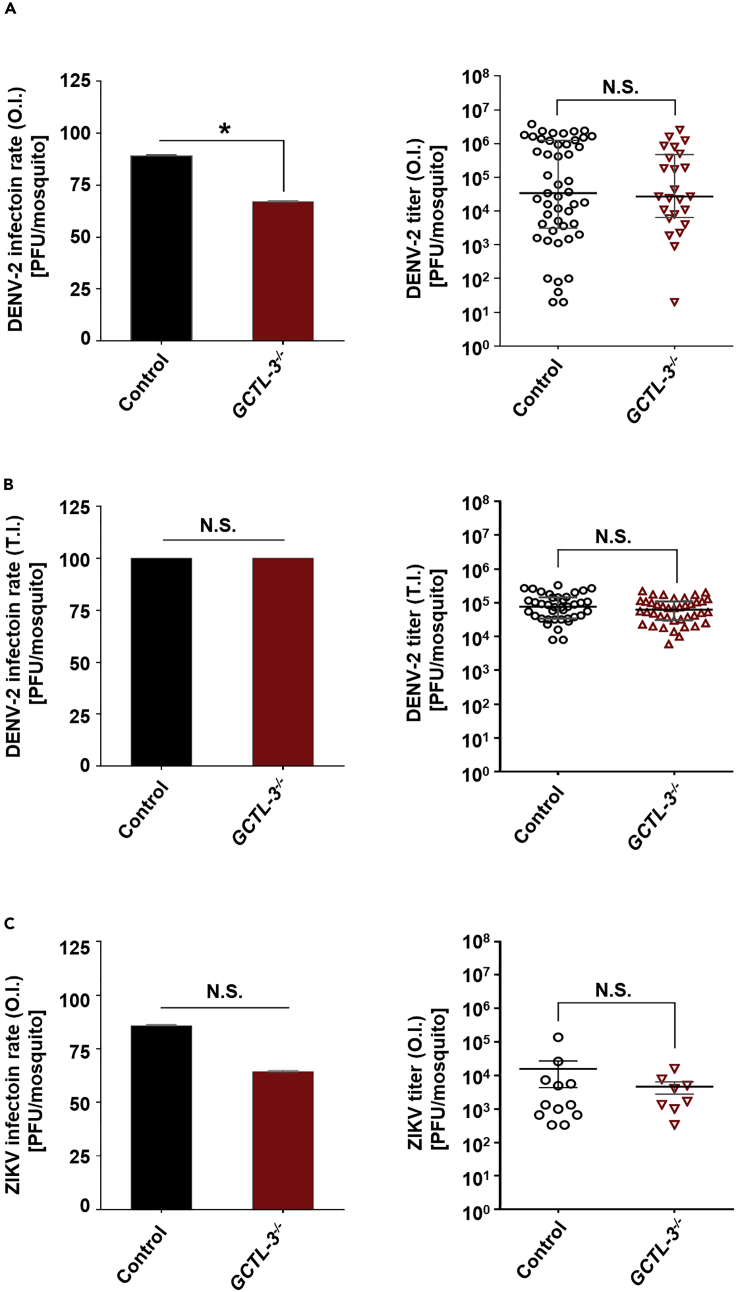
DENV-2, but Not ZIKV, Infection Rate Was Reduced in *GCTL-3*^*−/−*^ Mosquitoes 7 Days Post-blood Meal (A–C) Infection rate of (A) DENV-2 and (C) ZIKV in control and *GCTL-3*^*−/−*^ mosquitoes 7 days post-blood meal and virus titer of (B) DENV-2 in control and *GCTL-3*^*−/−*^ mosquitoes 7 days post-thoracic injection tested via plaque forming assays in BHK-21 or Vero cells. Sample sizes (DENV-2 for oral infection): Control = 55; *GCTL-3*^−/−^ = 36. Sample sizes (DENV-2 for thoracic injection): Control = 39; *GCTL-3*^−/−^ = 40. Sample sizes (ZIKV for oral infection): Control = 14; *GCTL-3*^−/−^ = 14. Data are represented as mean ± SD for infection rate and represented as median with interquartile range for virus titer. Asterisks represent significant differences between the genotypes (Mann-Whitney test; ∗p < 0.05. For infection rate, p = 0.0142 for (A); p > 0.9999 for (B); p = 2087 for (C). For virus titer, p = 0.8179 for (A); p = 0.2062 for (B); p = 0.7185 for (C); raw data related to Figure 2 were indicated in Supplemental Information, Data S1). N.S., no significant difference.

To investigate whether GCTL-3 plays a role in arbovirus infection, we first challenged *GCTL-3*^*−/−*^ mutants with DENV-2 via an artificial membrane blood feeding system and examined virus titers 7 days after this blood meal using plaque formation assay. We found a reduced infection rate for *GCTL-3*^*−/−*^ mutants compared with controls, with 89% of the control mosquitoes being infected when compared with 67% of mutants (Mann-Whitney test; p = 0.0142). However, we found no significant difference between the groups in terms of viral titer whether it is challenged via oral infection (with median titers of 2.7 × 10^4^ plaque-forming unit [PFU]/mL for mutants and 3.4 × 10^4^ PFU/mL for controls, Mann-Whitney test; p = 0.8179, Figure 2A) or via thoracic infection (with median titers of 6.3 × 10^4^ PFU/mL for mutants and 7.6 × 10^4^ PFU/mL for controls, Mann-Whitney test; p = 0.2062, Figure 2B), as detected via plaque assay.

To verify if *GCTL-3* knock-out affected viral titers of other members of the family Flaviviridae, we challenged *GCTL-3*^*−/−*^ mutants with 1 × 10^6^ PFU/mL ZIKV via oral infection. No significant differences were found between mutants and controls in terms of infection rate (64.3% and 85.7%, respectively; Mann-Whitney test; p = 0.2087) or viral titer (with median titers of 2.8 × 10^3^ PFU/mL for mutants and 1.3 × 10^3^ PFU/mL for controls, Mann-Whitney test; p = 0.7185, Figure 2C).

### Reduced Commensal Microbiota Populations in *GCTL-3*^*−/−*^ Midgut

**Figure 3:**
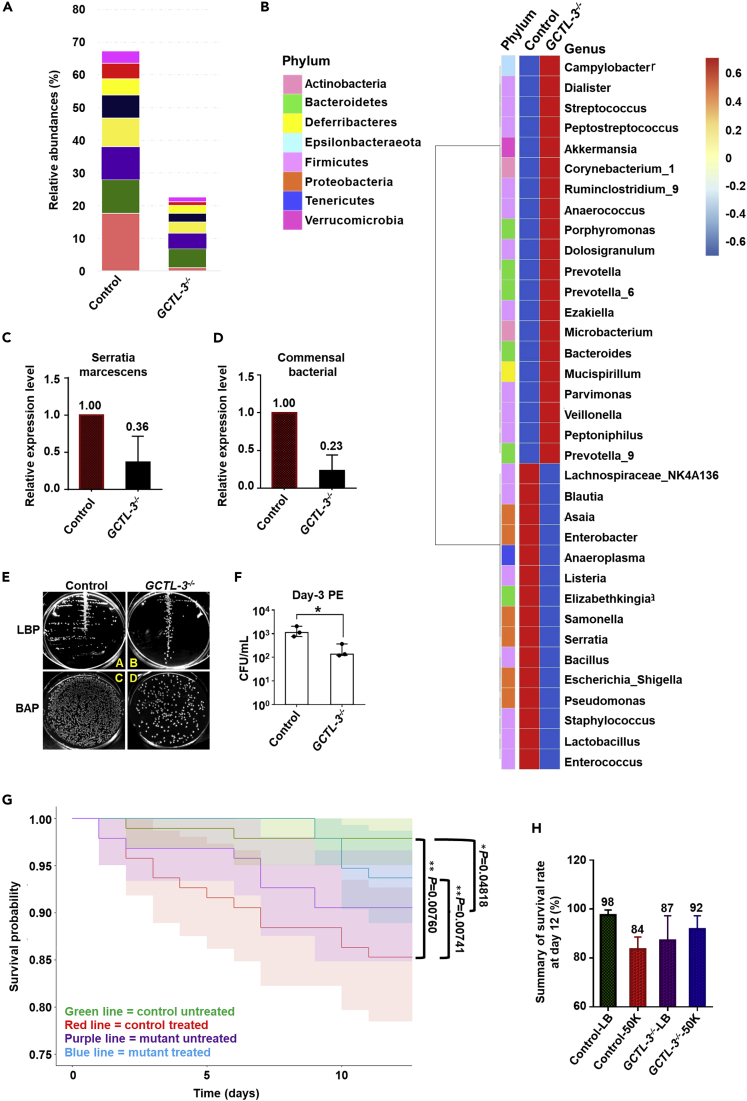
Reduced Colonization of *A. aegypti mosGCTL-3*^*−/−*^ Midgut by Gut Bacteria (A and B) 16S amplicon sequencing data from control and *GCTL-3*^*−/−*^ mosquitoes. Sample sizes: all groups = 15. (C and D) RT-PCR data indicated (C) commensal bacteria and (D) *S. mar* in *GCTL-3*^*−/−*^ mosquitoes. Sample size: each group = 10; data are represented as mean ± SD. (E and F) An abundance of bacteria detected in four mosquito whole bodies. *GCTL-3*^*−/−*^ mosquitoes were found to have fewer bacteria than controls. Data are represented as median with interquartile range. Unpaired t test was applied; ∗p < 0.05. (G) Mosquito survival curves following oral infection with *S. marcescens*; bacterial infection resulted in reduced mortality rates in mutants compared with controls. There were significant differences between genotypes (p = 0.0481) and treatment groups (p = 0.0076). The total sample size of each group was 95. Asterisks represent significant differences between the genotypes (Cox proportional hazards model; ∗p < 0.05, ∗∗p < 0.01). The solid line represents the median estimate, and the zones represent the confidence intervals, with the upper and lower bounds based on a Kaplan-Meier estimate. (H) Summary of survival rate at day 12 following bacteria challenge. Data are represented as mean ± SD. 50K, treatment with 50,000 CFU/mL of *S. marcescens;* BAP, blood agar plate LB, lysogeny broth; LBP, lysogeny broth plate. See also Table S5.

GCTLs play a substantial role in facilitating colonization of commensal bacteria in the mosquito midgut (Pang et al., 2016). To address whether the knock-out of *GCTL-3* affected the mosquito gut commensal microbiome, we used 16S amplicon sequencing to investigate *GCTL-3*^*−/−*^ gut microbiota populations. We found that *GCTL-3*^*−/−*^ mosquitoes had lower overall microbiota populations than controls, with reductions in eight operational taxonomic unit clusters (Figure 3A), as well as increases in two clusters (20% *Dolosigranulum* and 18% *Corynebacterium* compared with controls; data not shown). Fifteen genera were found to have lower levels in *GCTL-3*^*−/−*^ mutants, including *S. marcescens* and *Salmonella*, common components of the midgut microbiome (Figure 3B). RT-qPCR data provided further evidence that *S. marcescens* abundance was reduced in *GCTL-3*^*−/−*^ when compared with controls (2.5 × 10^2^ colony-forming unit [CFU]/mL and 1.3 × 10^3^ CFU/mL respectively) (Figure 3C). In line with these, *GCTL-3*^*−/−*^ midgut were found to have reduced bacterial DNA levels (Figure 3D) and fewer bacterial colonies than control mosquitoes (Figure 3E), as determined via colony forming assay (1.3 × 10^3^ CFU/mL for control and 2.5 × 10^2^ CFU/mL for *GCTL-3*^*−/−*^) (Figure 3F).

Given the previously reported role of *S. marcescens* in facilitating DENV infection, and the adverse effects of this bacterium on other model organisms (Grimont and Grimont, 1978; Kurz et al., 2003; Patil et al., 2011; Wu et al., 2019), we tested the effect on the lifespan of control and mutant mosquitoes when challenged with *S. marcescens* via oral infection. It was observed that 12 days after infection, the survival rates for controls drop significantly from 98% to 85%, whereas the survival rate of *GCTL-3*^*−/−*^ mutant increased slightly from 91% (untreated) to 94% (treated), consistent with the deleterious effects of *S. marcescens* on mosquitoes. Furthermore, we found a significant interaction between genotype and treatment, indicating that exposing mutants to *S. marcescens* resulted in a significantly different effect on mortality than when exposing controls (p < 0.01) (Figure 3G, Supplemental Information, Table S5).

### Activation of JAK/STAT, IMD, Toll, and AMPs Signaling Pathways in *GCTL-3*^*−/−*^ Mutant Mosquitoes

As upregulation of CTLDcps plays a role in facilitating viral entry and replication via activation of the Toll, IMD, or JAK/STAT pathways and induced AMPs (Jupatanakul et al., 2017; Kingsolver et al., 2013; Xi et al., 2008), we investigated the effect of *GCTL-3* knock-out on these signaling pathways. We found that many lectins became activated 1 to 3 days following a blood meal, including CTL-15, CTL-19, CTLGA-3, CTLGA-5, and GCTL-3 (Figure S1I, Supplemental Information, Table S6).

**Figure 4:**
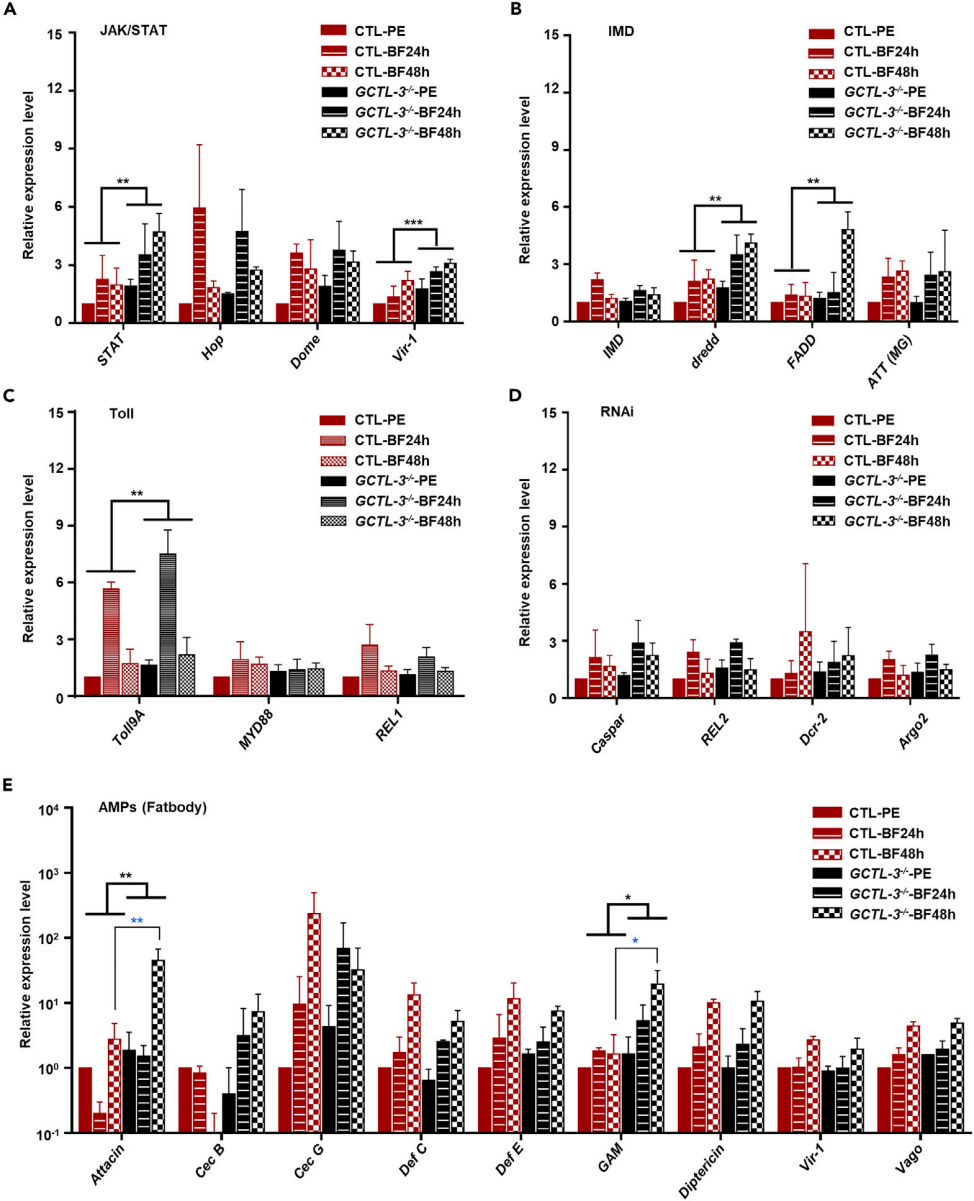
Knock-out of *GCTL-3* Causes a Change in the Regulation of JAK/STAT and AMP Signaling Pathway Genes (A–E) Midguts and fat bodies were dissected and collected from 7-day-old control and *GCTL-3*^*−/−*^ mosquitoes 24 and 48 h after blood feeding. Gene expression was normalized to the *A. aegypti* housekeeping gene RpS7. *GCTL-3*^*−/−*^ mosquitoes showed higher expression levels at marked time points in the (A) JAK/STAT, (B) IMD, (C) Toll, (D) RNAi, and (E) AMPs pathways. Sample sizes: all groups = 10. Data are represented as mean ± SD. Black asterisks represent significant differences between the genotypes (two-way ANOVA; ∗p < 0.05, ∗∗p < 0.01, ∗∗∗p < 0.001; exact p values for each comparison can be found in Supplemental Information, Table S7 and Data S2) and blue asterisks represent significant differences between genotypes at a particular time. BF, blood feed; PE, post-eclosion. See also Tables S7 and S8.

Before a blood meal, *GCTL-3*^−/−^ mosquitoes showed elevated expression levels of *STAT* (AAEL009692) and *Vir-1* (AAEL000718), which are signaling components of the JAK/STAT pathway (Figure 4A, Supplemental Information, Table S7). Following a blood meal, however, these differences broadly disappeared, although *STAT* levels were still significantly greater in mutants 48 h post-blood meal. Taken together, these results suggest an activation of JAK/STAT signaling in *GCTL-3* mutants following blood meal consumption.

In addition, 48 h after a blood meal, *GCTL-3* knock-out also resulted in increased expression of *dredd* (AAEL014148) and *FADD* (AAEL001932), both of which are components of the IMD pathway (Figure 4B, Supplemental Information, Table S7). The uptake of a blood meal did not seem to affect regulation of either the Toll or RNAi pathway in *GCTL-3* mutants (Figures 4C and 4D, Supplemental Information, Table S7). However, blood meal provision resulted in significantly higher expression levels of *Attacin* (*ATT*, AAEL003389) and *Gambicin* (*GAM*, AAEL004522), but not *Defensin E* (*Def E*, AAEL000611), in mutants 48 h after the blood meal. All comparisons were analyzed using two-way ANOVA (Figure 4E, Supplemental Information, Table S7); full details of the ANOVA values related to Figure 4 are recorded in Supplemental Information Table S8.

Collectively, the data shows an elevated immuno-response in *GCTL-3* mutants compared with controls after consumption of a blood meal. A previous study by Ramirez et al. found that transcript abundance of mosquito AMP genes changed 2 days after mosquito midgut bacteria were introduced (Ramirez et al., 2012); this indicates that *GCTL-3* not only influences viral dynamics but also regulates gut homeostasis and innate immune response following blood meal uptake, suggesting that *GCTL-3* influences multiple *in vivo* functions.

### *GCTL-3* Knock-out Resulted in Defects in Mosquito Fertility and Fecundity

**Figure 5:**
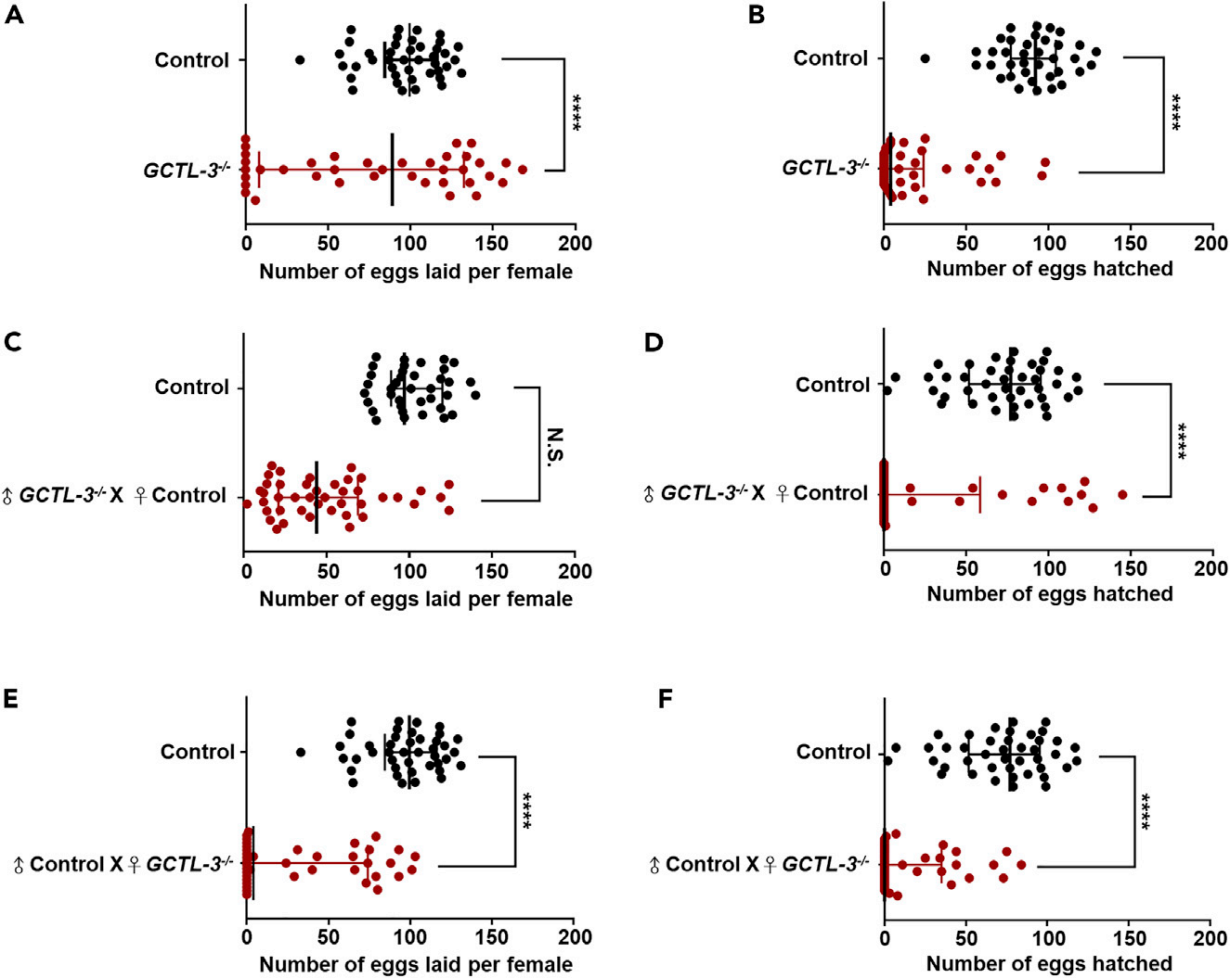
*GCTL-3*^*−/−*^ Mutants Show Reduced Oviposition and Egg Hatch Rates Compared with Controls (A and B) (A) Number of embryos and (B) number of hatched larvae for *mosGCTL-3*^*−/−*^ mutants and controls. Sample sizes: control = 37; *GCTL-3*^*−/−*^ = 43. (C–F) (C and D) Mutant male (N = 38) and (E and F) female (N = 39) mosquitoes were backcrossed to control mosquitoes carrying mutations for *A. aegypti GCTL-3* genes, and the number of embryos and larvae in *GCTL-3*^*−/−*^ mutant and control progeny in the subsequent generation were recorded. Sample sizes: control = 42; *GCTL-3*^*−/−*^ = 42. Data are represented as median with interquartile range. Asterisks represent significant differences between the genotypes (Mann-Whitney test; ∗∗∗∗p < 0.0001. p = 0.5995 for (C); p < 0.0001 for (A, B, and D–F); Supplemental Information, Data S3).

To better understand the relationship between immunity and reproduction, we next investigated the effect of upregulation of the JAK/STAT and AMPs pathway and altered gut microbiota populations arising from *GCTL-3* knock-out on mosquito fecundity and fertility. The numbers of embryos laid per female and egg hatching rate were both significantly reduced in *GCTL-3*^−/−^ mosquitoes when compared with controls; female controls produced approximately 100 embryos each, around double that of mutants, whereas hatching rate was reduced from 90% to 40% in mutants (Mann-Whitney test; p < 0.0001 for both embryo number and hatched larvae; Transparent Methods; Figures 5A and 5B). *GCTL-3* knock-out also caused embryo melanization and abnormally shaped ovarioles in mutants (Supplemental Information, Figures S4A and S4B, Data S7); although melanization plays an important role in the invertebrate defense system, here it likely led to a significant increase in the number of non-viable eggs (Shin et al., 2011; Zou et al., 2010). We found that the *GCTL-3* knock-out caused defects in mosquito oviposition that were not PPO3-dependent (Supplemental Information, Figure S4C).

To address whether decreases in fecundity and fertility were due to defects in either male or female mosquitoes (or both), we back-crossed *GCTL-3*^*−/−*^ male or female mosquitoes with wild-type mosquitoes. We found no differences between controls and *GCTL-3*^*−/−*^ males in terms of fecundity (Mann-Whitney test; p = 0.5995; Figure 5C) but identified a significant reduction in *GCTL-3*^*−/−*^ male fertility (Mann-Whitney test; p < 0.0001; Figure 5D), indicating that there may be a reduction in sperm count in mutant males. We further found that *GCTL-3*^*−/−*^ females exhibited strong reductions both in fecundity and fertility, by counting the eggs of mosquitoes and the number of larvae hatched in next generation (fecundity of controls = 37; fecundity of mutants = 43; Mann-Whitney test; p < 0.0001; Figures 5E and 5F). We also checked for differences in physiology, which included body weight, body size, wing size, host-seeking behavior, and survival rates of mosquitoes and found that female mutant lifespan was significantly shorter than that of controls (Supplemental Information, Figure S5 and Data S8).

### Germline Abnormalities in the Ovaries of *GCTL-3*^−/−^ Mutants and the Loss of *GCTL-3* in the Mosquito Midgut Activated Apoptotic Signaling Pathways

**Figure 6:**
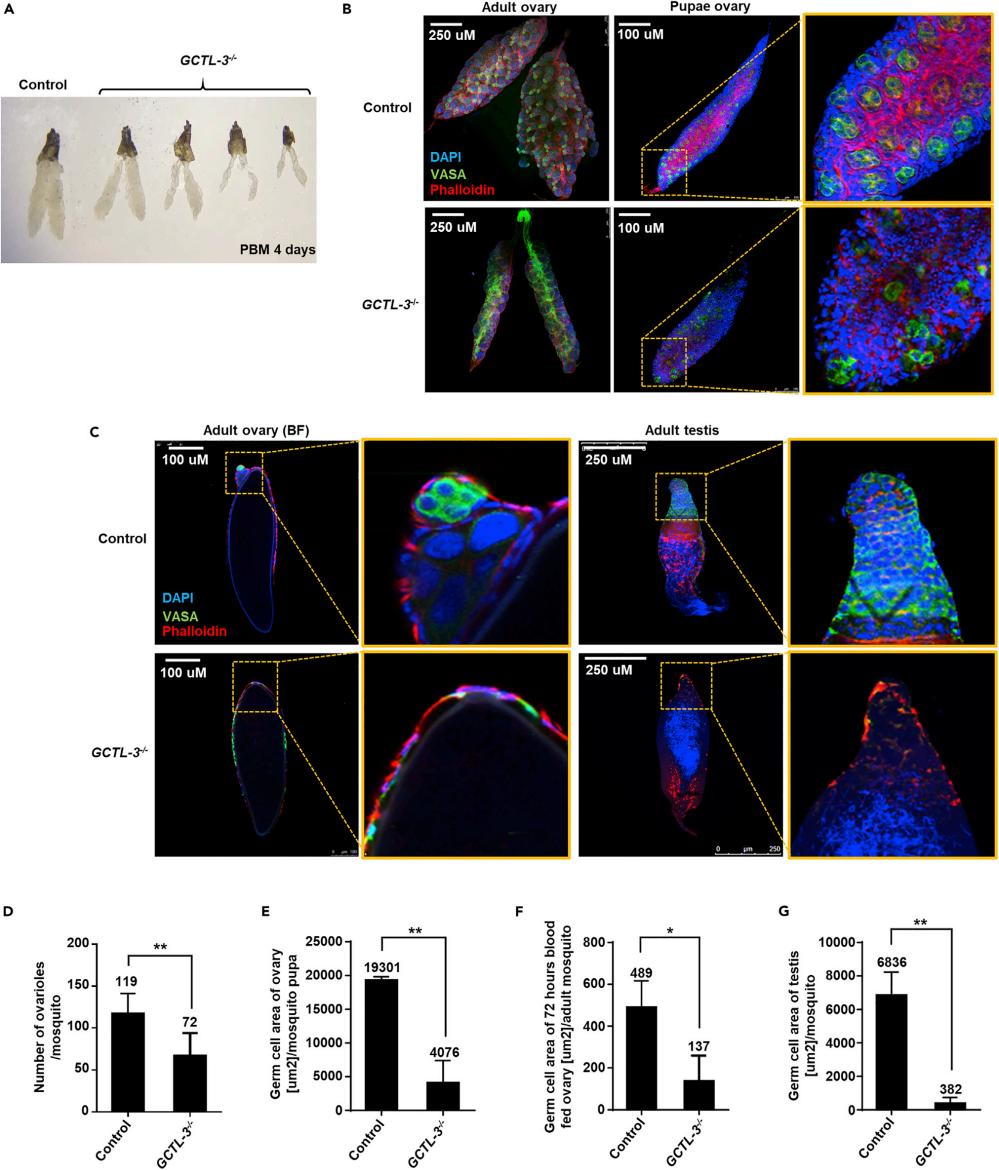
VASA Expression in *GCTL-3*^−/−^ Ovaries (A) Comparison of ovaries of 5- to 7-day-old control (left) and *GCTL-3*^*−/−*^ (right) mosquitoes. Sample sizes: all groups = 7. (B and C) VASA expression in 5- to 7-day-old control and *GCTL-3*^*−/−*^ female (B) non-blood-fed adult and pupae ovaries as well as (C) 72-h post-blood-fed ovaries and adult male testes. (D–G) Quantification of immunostaining across three samples in (D) non-blood-fed female ovaries, (E) pupae ovaries, (F) 72-h post-blood-fed ovaries, and (G) male testes. Anti-VASA was used as a primary antibody (1:500), and Alexa Fluor 488 dye was used as a secondary antibody (1:500) along with DAPI and phalloidin staining to mark the cell nuclei and cytoskeletons. Data are represented as median with interquartile range. Stars represent significant differences between the genotypes (Mann-Whitney test; ∗p < 0.05, ∗∗p < 0.01. p = 0.0089 for (D); p = 0.0014 for (E); p = 0.0262 for (F); p = 0.0015 for (G); Supplemental Information, Data S4). BF, blood feed.

To better understand the mechanisms underlying the reduced fertility of *GCTL-3*^−/−^ mosquitoes, we examined mutant ovaries 4 days after a blood meal. *GCTL-3*^−/−^ mosquitoes were found to have significantly fewer ovarioles than controls (Mann-Whitney test; p = 0.0250; Figures 6A and 6D), suggesting defects in early germ line development.

We therefore investigated germline development in control and *GCTL-3*^−/−^ pupae via immunohistochemistry using an anti-*Aa*. VASA antibody. VASA is an evolutionarily conserved germ cell maker found in many different organisms (Castrillon et al., 2000; Gustafson and Wessel, 2010; Raz, 2000). VASA immunostaining also indicated a reduction of signal in *GCTL-3*^−/−^ pupae gonads (Figure 6B right and 6E) and ovaries compared with controls (Figure 6B left). Furthermore, a significant fraction of blood-fed mutant ovarioles did not contain a germarium, the anterior region of the ovariole likely to contain germline stem cells (Figure 6C left and 6F), suggesting that *GCTL-3* contributes to mosquito germline development. We also observed increased expression of the apoptosis marker cleaved-caspase-3 in *GCTL-3*^−/−^ ovaries (Supplemental Information, Figures S6A and S6C, Data S9). The reduction in germ cells and increased levels of apoptosis are thus likely the cause of the reduced number of eggs produced by mutant females. Similarly, many *GCTL-3*^−/−^ testes were less organized and exhibited a reduced VASA signal (Figure 6C right and 6G, Data S9) as well as an increased cleaved-caspase-3 signal (Supplemental Information, Figures S6B and S6D, Data S9). Furthermore, some *GCTL-3*^−/−^ testes were found to lack VASA-expressing germ cells (Figure 6B right and 6C, Data S9).

**Figure 7:**
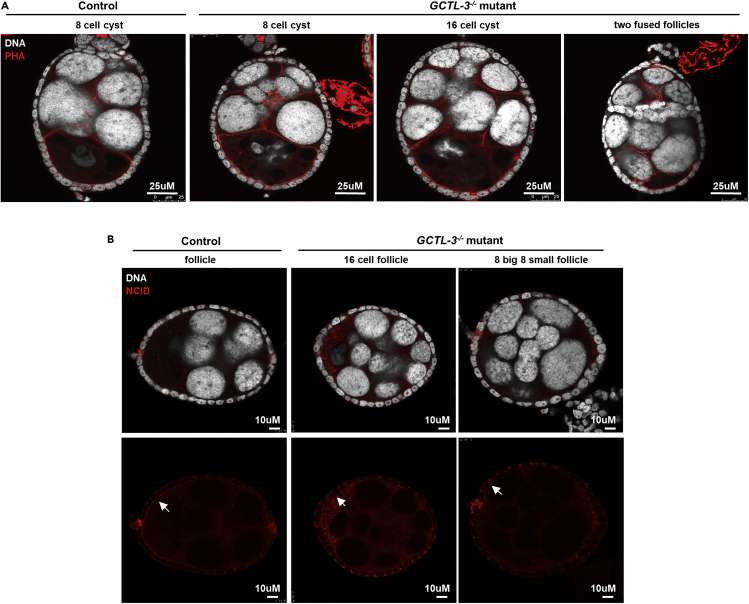
Defects in *GCTL-3*^*−/−*^ Follicles (A) Left, a control follicle containing seven nurse cells and one oocyte; right, a *GCTL-3*^*−/−*^ follicle containing various numbers of nurse cells and oocytes. (B) NICD (labeled by arrows) is mainly localized on the apical side of follicular cells in control mosquitoes (left); the extent of this localization is reduced in *GCTL-3*^*−/−*^ follicular cells. DNA was visualized using Hoechst staining. See also Table S9.

In addition to its role during early germline development, *GCTL-3* also seems to serve a vital function in regulating mosquito oogenesis. In control mosquitoes, germline stem cells/progenitors undergo three rounds of synchronized divisions to produce an 8-cell cyst (7 nurse cells and 1 oocyte) with three ring canals connecting the oocyte to the nurse cells, whereas the *Drosophila* germline stem cell undergoes four rounds of synchronized divisions to produce a 16-cell cyst (Spradling, 1993). However, 17.27% of *GCTL-3*^−/−^ follicles analyzed contain a 16-cell cyst (0.95% in control), indicating four rounds of germline cell divisions. Consistent with one extra round of germline cell division in these follicles, these follicles contained 15 polypoid nurse cells and one oocyte (Figure 7A). Furthermore, the oocyte was connected to the nurse cells via four ring canals instead of the usual three ring canals found in a normal 8-cell follicle (not shown).

We found that *GCTL-3*^−/−^ ovaries also exhibit defective encapsulation in terms of individualization of germline cysts. In control mosquito ovaries, each germline cyst is encapsulated by a layer of somatic cells upon exit of germarium to form a germline follicle. Each follicle is separated from neighboring follicles by a stack of interfollicle stalk cells. In *GCTL-3*^−/−^ ovaries, however, 22.87% of follicles were identified as compound follicles, containing fused follicles with various germ cells and lacking interfollicle stalk cells (Supplemental Information, Table S9).

Previous reports indicated that during *Drosophila* oogenesis, defects in the Notch pathway can produce similar encapsulation defects (Ruohola et al., 1991; Xu et al., 1992). We therefore examined Notch localization in mosquito follicular cells. Similar to its localization in *Drosophila* follicular cells, Notch (recognized by an anti-*Drosophila* NICD antibody) was expressed and mainly localized on the apical domain (facing the germline side) of follicular cells in control mosquitoes. We found weak apical localization in *GCTL-3*^−/−^ follicular cells (Figure 7B), suggesting that *GCTL-3* may play a role in regulating Notch apical localization, which may be the cause of the defective encapsulation. We also found that cleaved-caspase-3 signal accumulated in *GCTL-3*^−/−^ midguts following a blood meal. This was clear from both qPCR (Supplemental Information, Figure S7A, Data S10) and immunostaining (Supplemental Information, Figure S7B) data and was not the case for control mosquitoes.

### *Attacin* and *Gambicin* Knock-down Partially Rescued Reductions in *GCTL-3*^*−/−*^ Fertility and Fecundity

Changes in expression levels of components of the AMPs immunological pathway have been found to significantly affect insect reproductive capabilities (Camaioni et al., 2018; Delhaye et al., 2016; Schwenke et al., 2016). Given the significant increase found for various elements of this pathway in *GCTL-3*^−/−^ mutants (Figure 4E), we hypothesized that reducing the expression of these elements may rescue female fecundity. As lower doses of dsRNA (of 1μg) were not effective to knock-down AMPs in *GCTL-3*^−/−^ mutants (data not shown), we instead used 1.5 μg dsRNA to knock-down *Attacin* and *Gambicin,* which we identified as significantly upregulated in mutants following a blood meal (Figure 4E), to assay the role of *GCTL-3* in the immunity to reproduction trade-offs.

**Figure 8:**
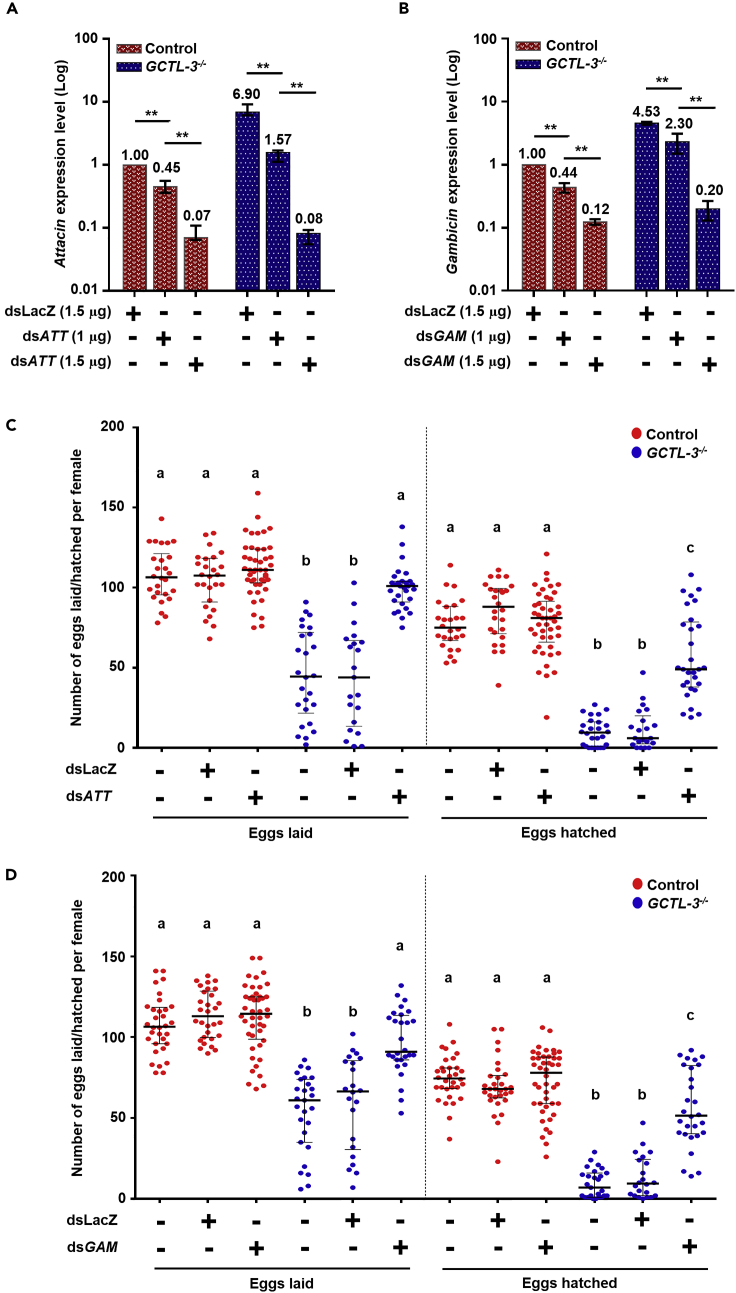
Attacin and Gambicin Knock-down Partially Rescued Reductions in *GCTL-3*^*−/−*^ Fertility and Fecundity (A and B) Change in (A) *Attacin* and (B) *Gambicin* expression levels for control and *GCTL-3*^*−/−*^ mosquitoes at different time points and injection states detected via reverse transcription-PCR. Data are represented as mean ± SD. (N = 6 for each group. Mann-Whitney test; ∗∗p < 0.01). (C and D) Egg (left) and larval hatch (right) counts for control (red) and *GCTL-3*^*−/−*^ (blue) mutants following no injection, dsLacZ injection, dsATT injection and dsGAM injection. Each point represents an egg/larval hatch count for an individual female. Sample sizes for *Attacin* testing: Control = 26; Control + dsLacZ = 26; Control + dsATT = 45; *GCTL-3*^*−/−*^ = 21; *GCTL-3*^*−/−*^ + dsLacZ = 26; *GCTL-3*^*−/−*^ + dsATT = 29. Sample sizes for *Gambicin* testing: Control = 32; Control + dsLacZ = 30; Control + dsGAM = 44; *GCTL-3*^*−/−*^ = 27; *GCTL-3*^*−/−*^ + dsLacZ = 22; *GCTL-3*^*−/−*^ + dsGAM = 28. dsLacZ/dsATT/dsGAM represents groups injected with double-stranded RNA for LacZ/*Attacin*/*Gambicin*. Data are represented as median with interquartile range. Different letters represent significant differences between groups (two-way ANOVA; adjusted p < 0.05). Exact p values for each comparison can be found in Supplemental Information, Table S11 and Data S5. See also Tables S10 and S11.

dsATT and dsGAM injection does not affect control egg laying rate, but restores *GCTL-3*^−/−^ mutant egg laying rate to control levels (two-way ANOVA; p < 0.05 respectively; Figures 8C and 8D left, Supplemental Information, Tables S10 and S11). dsATT and dsGAM injections do not, however, restore *GCTL-3*^−/−^ mutant larval hatching to control levels, although they still significantly increase the number of larvae hatching compared with control and dsLacZ injections (two-way ANOVA; p < 0.05 for all comparisons). No differences were found between any control groups (two-way ANOVA; p > 0.05) (Figures 8C and 8D right, Supplemental Information, Tables S10 and S11).

## Discussion

Recent years have seen major breakthroughs in mosquito gene editing techniques, ranging from the initial demonstration of CRISPR/Cas9 in *A. aegypti* to the knock-out of kynurenine hydroxylase (*kh*) and dopachrome conversion enzyme (*yellow*), thus creating mosquito white eye (loss of pigment) and yellow body mutants, to the establishment of transgenic germline-specific Cas9 *A. aegypti* founder strains (Kistler et al., 2015; Li et al., 2017; Liu et al., 2018; Yang et al., 2019). Basu et al. and Li et al. previously used the CRISPR-Cas9 system to generate site-specific mutations in *A. aegypti* by injecting *in vitro*-transcribed sgRNA that used a homology-directed repair (HDR) technique. Here, we used the *Aedes* U6 promoter to drive sgRNA expression *in vivo* and co-injected the U6 promoter-driven sgRNA template with the HDR construct plasmid.

By applying this methodology, we were able to knock-out a member of the CTL family, *GCTL-3*, to investigate the resource trade-offs that occur in female mosquitoes following pathogen infection. Previous mosquito work on reproductive/immunological trade-offs has mainly focused on *Anopheles gambiae*. CRISPR/Cas9 methodologies have been used in that study to generate a mosaic gamma-interferon-inducible lysosomal thiol reductase (mos*GILT*) mutant line, which showed both defects in ovary development and an anti-*Plasmodium* phenotype (Yang et al., 2019). No such mutants have previously been generated in *A. aegypti,* however, and only the general mechanisms underlying these trade-offs are known.

We confirmed that AAEL000535 was a member of the CTL family based both on previous work on *A. aegypti* CTL and a recent article by Pascini et al., who provided information regarding the reassembled coding sequences of AAEL000535 and AAEL029058 (Pascini et al., 2020). This information indicated that in terms of DNA/RNA sequences, AAEL000535 and AAEL029058 are the same locus and belong to the CTLs. Based on a Vectorbase alignment of the sequences, we believe AAEL000535 may either be the same gene or an alternative splicing form of AAEL029058 that lacks the additional putative sequence on the N-terminal region of the protein.

Prior publications have discussed the role played by various signaling pathways, including the Toll, IMD, JAK-STAT, and RNAi pathways, in limiting pathogen propagation following infection (Kumar et al., 2018). Mosquito commensal microbiota also play a vital role in DENV immunological responses via activation of the Toll immune pathway, whereas increased expression of JAK-STAT signaling components in the mosquito fat body has been shown to inhibit DENV infection in the midgut and the salivary glands (Jupatanakul et al., 2017; Xi et al., 2008). Moreover, each mosquito tissue performs specific antiviral strategies (Cheng et al., 2016). Each of these mechanisms is likely to lead to a reduction in mosquito reproductive capabilities due to resource limitations. CTLDcps expression level varies significantly between males and females, as well as across different developmental stages and parts of the mosquito body (Adelman and Myles, 2018). We thus investigated expression levels of *GCTL-3* in different male and female *A. aegypti* body parts, including the head, thorax, fat body, ovary, and testis. Expression levels in the head were found to be higher than in any other body part for both sexes (Supplemental Information, Figures S1G and S1H), suggesting that *GCTL-3* may play a role in regulating brain function.

DENV-2 (NGC strain) has been reported to be particularly virulent and the cause of many severe dengue outbreaks (Wang et al., 2016; Williams et al., 2014; Yung et al., 2015). Most research articles (Molina-Cruz et al., 2005, Salazar et al., 2007, Sanchez-Vargas et al., 2018, Sri-In et al., 2019, Tree et al., 2019) have used DENV-2 for proof-of-principle experiments. Here we utilized DENV-2 NGC, the most commonly used strain. In this study, we found that *GCTL-3*^−/−^ *A. aegypti* mutants showed a reduction in DENV-2 infection rate and altered expression levels for various components of key signaling pathways, indicating that *GCTL-3* is involved in the JAK-STAT, IMD, Toll pathways, and AMPs activation. In the previous article by Liu et al. (2014), RNAi knock-down of *GCTL-3* decreased DENV replication; here, however, knock-out of *GCTL-3* did not lead to a reduction in virus titer. A median decrease in viral titer of 60% could have a significant effect on the resulting infection rate (Buchman et al., 2019; Souza-Neto et al., 2019); verification of *GCTL-3* mutant infection rates is therefore a necessary next step.

Following a blood meal, the JAK-STAT pathway became activated and downstream AMPs expression levels were altered. We found that *GCTL-3* knock-out led to a reduction in the number of gut microbiota, suggesting that *GCTL-3* plays a role in promoting gut microbiota homeostasis. This may be related to significant increases in expression levels seen for two AMPs, *Gambicin* and *Attacin*, in *GCTL-3*^−/−^ mutants. The regulation sites of the *Gambicin* promoter region have been identified, and *Gambicin* can be induced by the IMD, Toll, and JAK-STAT pathways via combinatorial regulation in *A. aegypti* Aag2 cells (Zhang et al., 2017). Furthermore, *Attacin* has been reported to combat Gram-negative bacterial infection in *Drosophila* (Wicker et al., 1990).

Mosquitoes are hematophagous insects that can obtain many pathogens via blood feeding; the first line of defense to these pathogens is therefore the intestinal tract, which includes the gut commensal microbiome. This microbiome can be highly diverse, with 21 bacterial species having been identified in the *A. aegypti* Rockefeller strain (Wu et al., 2019). From our 16S sequencing data and CFU assay results, it is clear that *GCTL-3* knock-out causes a change in gut bacteria homeostasis. This is particularly relevant in the case of *S. marcescens*, which has been identified as the main bacterium in control mosquito midguts and can enhance viral dissemination in mosquitoes (Wu et al., 2019). In our study, loss of *GCTL-3* resulted in a corresponding loss of *S. marcescens* from the mosquito midgut, which may be the cause of the decreased virus infection rate found in mutants. Formation of a microbiota-induced peritrophic matrix has previously been reported as preventing pathogen infection via regulation of midgut homeostasis in *Anopheles* mosquitoes (Rodgers et al., 2017). Further research into expression levels of *Sm*Enhancin and structure formation of the peritrophic matrix in *A. aegypti* is thus of great interest.

Gut homeostasis plays an important role in determining developmental rate and reproductive output in many species (Elgart et al., 2016; Leitao-Goncalves et al., 2017). Correspondingly we found that *GCTL-3* mutants, whose gut microbiota populations were severely reduced compared with controls, exhibited clearly defective ovaries and testes as well as shortened lifespans. We also noticed defects in germline development; in controls, 93.3% of germline follicles were normal (i.e., contained seven nurse cells and one oocyte [total = 393]), whereas only 50.6% of germline follicles were found to be normal in *GCTL-3* female mutants (total = 411) (Supplemental Information, Table S9). Knock-out of *GCTL-3* in *A. aegypti* thus appears to cause similar germline developmental defects as removal of the gut bacteria of *Drosophila*. CTLs thus play an important role in germ line development and reproduction.

Uptake of a blood meal by a female mosquito results in the production of two signals: a direct signal to the fat body, activated by yolk protein precursor (YPP) gene expression, and an indirect signal from the midgut to the brain. The latter signal activates medial neurosecretory cells to release a peptide hormone, ovarian ecdysteroidogenic hormone (OEH), which then produces ecdysone in the fat body to activate the steroid hormone, 20-hydroxyecdysone (20E). 20E in turn activates YPP gene expression (Raikhel et al., 2005). In this study, the highest levels of *GCTL-3* mRNA were found in the mosquito head, suggestive of a role for *GCTL-3* in modulating brain function.

Production of AMPs has been found to alter female mosquito response to pathogens (Schwenke et al., 2016). Here we used dsRNA to knock-down two components of the AMPs pathway, *Attacin* and *Gambicin*, which were found to be significantly upregulated in mutants compared with controls following consumption of a blood meal. We found that suppression of *Attacin* and *Gambicin* could rescue in part the reproductive defects of mutants, implying that *Attacin* and *Gambicin* may play important roles in *GCTL-3*-mediated reproductive processes.

Silencing of *AaNotch* and *AaJNK* results in significant reductions of female mosquito fecundity and fertility (Chang et al., 2018). Our data indicate a reduction in Notch signal intensity or alterations in localization in *GCTL-3* mutant ovaries 24 h post-blood meal, implying that CTLs may influence Notch localization and activity during reproductive processes.

Activation of apoptosis is a hallmark of host cell protection against pathogenic infection; this is executed by the family of cysteinyl proteases that includes caspase 3, whose activation is a crucial event for efficient influenza virus propagation (Thornberry and Lazebnik, 1998; Wurzer et al., 2003). Previous reports have indicated that the denudation of germline development is sufficient to extend the lifespan in *C. elegans* and *Drosophila* (Flatt et al., 2008; Yunger et al., 2017). In mosquitoes, the role of *GCTL-3* in affecting longevity is not clear. Here, we used a cleaved-caspase-3 antibody to address germline defects in *GCTL-3* mutants and identified up-regulated apoptotic signals. This could thus result in ovary defects and inhibit viral load in the mosquito midgut. *Michelob_x* (*Mx*) and *IMP*, two IAP antagonists involved in the apoptosis pathway, act on both initiator and effector caspases (Wang and Clem, 2011). Our data showed that loss of *GCTL-3* also resulted in caspase-3 activation after a blood meal, suggesting that *GCTL-3* may either introduce DIAP1 to the midgut or bind with *Mx* and/or *IMP* to protect *DIAP1* from degradation. Either mechanism would result in inhibition of apoptosis in the mosquito midgut.

Loss of *GCTL-3* caused activation of the genes *Hop*, *Dome,* and *STAT*, all of which play a role in the JAK-STAT pathway post-eclosion, as well as activation of the downstream gene *Vir-1* 24 48 h after a blood meal. Knock-out of *GCTL-3* also activated the IMD pathway, which represents another innate immunity defense mechanism. In *Drosophila*, the FADD (DmFADD) and caspase-8 homologs (DREDD) can associate with IMD to form a multimeric complex (Georgel et al., 2001). Here we found that post-eclosion and 48 h post-blood meal FADD and DREDD, in addition to *Attacin*, *Gambicin,* and *Defencin E*, were also activated in *GCTL-3*^*−/−*^ mutant mosquitoes. This pathway may also lead to upregulation of apoptosis markers and block DENV and ZIKV infections.

Finally, whereas many insect studies have identified negative correlations between up/down-regulation of immunological and reproductive pathways, few have determined the mechanisms, or components of these mechanisms, which modulate resource distribution (McKean et al., 2008). In *Drosophila melanogaster*, upregulation of IMD and JNK signaling has been reported to downregulate insulin-like growth factor signaling and thus egg production; 20-hydroxyecdysone and juvenile hormone are also thought to be involved in this pathway (DiAngelo et al., 2009; Schwenke et al., 2016). Here, we found increased expression levels of several components of both signaling pathways, suggesting that this pathway may be conserved in *A. aegypti*. Generation of further knock-out mutants for other members of the lectin family could help to precisely identify the role they play in influencing the balance between reproductive and immunological systems.

In summary, we here established a mutant *A. aegypti* line and investigated the important relationship between CTLs and arbovirus infection. The observed reductions in virus infection rate are likely the result of changes in the gut microbiome, providing further evidence to the key role played by microbiota in infection rate within the mosquito itself. CTLs not only play a vital role in mosquito immune responses and gut homeostasis but also seem to have important functions in germline development and life span determination. A better understanding of the links between reproduction and immune response as mediated via the lectin family should provide new information regarding insect resource allocation processes.

### Limitations of the Study

Based on our alignment, we believe that AAEL000535 is the truncated form of AAEL029058 lacking the N terminal. According to Vectorbase, AAEL029058 has an additional putative sequence on the N-terminal region of the protein belonging to the coding sequence. Given that the start codon is usually ATG (Methionine) for eukaryotic coding sequences, and that alternate start (non-ATG) codons are highly rare in eukaryotic genomes, there is insufficient evidence currently available to clarify which is the correct start codon for AAEL029058. Clarifying the full-length sequence of this gene is therefore important for validation purposes. Furthermore, testing whether the reduction in viral titer leads to a decrease in viral transmission rate would also provide valuable additional information.

### Resource Availability

#### Lead Contact

Further information and requests for resources and reagents should be directed to and will be fulfilled by the Lead Contact, Chun-Hong Chen (chunhong@gmail.com).

#### Materials Availability

Materials generated in this study are available from the Lead Contact with a completed Materials Transfer Agreement.

#### Data and Code Availability

The published article includes all data generated or analyzed during this study.

## Methods

All methods can be found in the accompanying Transparent Methods supplemental file.

## Supplemental Information

Document S1. Transparent Methods, Figures S1–S7, and Tables S1–S7 and S9–S11

Table S8. ANOVA Values for Comparisons between Controls and Mutants for Immune Pathway Components, Related to Figure 4

Document S2. Data S1–S10. Raw Data Included in This Study

## Footnotes

Supplemental Information can be found online at https://doi.org/10.1016/j.isci.2020.101486.
