# Supplementary material for: C-Type Lectins Link Immunological and Reproductive Processes in Aedes aegypti
Source: iScience. 2020 Aug 21;23(9):101486. doi: 10.1016/j.isci.2020.101486 (PMC7481239; doi:10.1016/j.isci.2020.101486)
Supplement: Document S1. Transparent Methods, Figures S1–S7, and Tables S1–S7 and S9–S11 [file mmc1.pdf]

**iScience, Volume *23***

## Supplemental Information

# C-Type Lectins Link

# Immunological and Reproductive

# Processes in *Aedes aegypti*

**Hsing-Han Li, Yu Cai, Jian-Chiuan Li, Matthew P. Su, Wei-Liang Liu, Lie Cheng, Shu-Jen Chou, Guann-Yi Yu, Horng-Dar Wang, and Chun-Hong Chen**

**Supplemental Data**

**Table S1. Primer designs for plasmid assembly, related to Figure 1**

**pBFv-*Aae*U6_*GCTL-3*-sgRNA vector**

| No | Name | Sequence |
|---|---|---|
| 1 | sgRNA of *GCTL-3* | 5'-GCCCAGTTGGTGTAGTTGACGGG-3' |
| 2 | *Ae*U6-gRNA-F1 | 5'-GCTTGATATCGAATTCCTATATAATTTAATTCCACTAGAGT-3' |
| 3 | *Ae*U6-gRNA-R1 | 5'-TAGCTCTAAAACGGAGACGAACTCCGTCTCCATTTCACTAC TCTTGCCTCTGCTCTTATA-3' |
| 4 | *Ae*U6-gRNA-R2 | 5'-TTTCAAGTTGATAACGGACTAGCCTTATTTTAACTTGCTATTT CTAGCTCTAAAACGGAG-3' |
| 5 | *GCTL-3*-sgRNA-F | 5'-AAATGCCCAGTTGGTGTAGTTGAC-3' |
| 6 | *GCTL-3*-sgRNA-R | 5'-AAACGTCAACTACACCAACTGGGC-3' |

**pCR2-TOPO-*GCTL-3*-attp-loxp-Pub-eGFP HR donor vector**

| No | Name | Sequence |
|---|---|---|
| 7 | *Ae*Pub-pr-F | 5'-GCTAGCTCTACCTAGGTATCTTTACATGTAGCTTGTGCATTG AATCC-3' |
| 8 | *Ae*Pub-pr-R | 5'-AGACCTCATGCGGCCGCGTTGAAATCTCTGTTGAGCAGAA AAA GAAACGAG-3' |
| 9 | *GCTL-3*-up-F | 5'-ATCCACTAGTGCTAGCTCAGTTTGCAATAAGCATTCAGCTT GTCTG-3' |
| 10 | *GCTL-3*-up-R | 5'-CTGACCTGGGCCCGGGGACGTGCTGTCCCGTTGCGTGCC ATATGAA-3' |
| 11 | *GCTL-3*-down-F | 5'-TCTGACCTGGGCATATGAACTACACCAACTGGGCGTTGAAT ATGCCG-3' |
| 12 | *GCTL-3*-down-R | 5'-TAGATGCATGCTCGAGACAATGGACGTCTTGTGTCCTACTT ATCTC-3' |

**Table S2. Efficiency of microinjection for generation of germline mutants, related to Figure 1 and Figure S2**

| **G0*** | | | | | **G1† adult** | |
|---|---|---|---|---|---|---|
| Embryos | Larvae | Survival | Fluorescent | adult | Visible eGFP | Germline mutant |
| **795** | **210** | **26%** | **168 (80%)** | **176** | **8 (4.5%)** | **2 (1.1%)** |

*G0: G0 generation., †G1: G1 generation

**Table S3. Primer designs for digital droplet PCR, related to Figure 1**

| No | Name | Sequence |
|---|---|---|
| **1** | **Rf-nk_ddPCR-F** | **5'-**CGTGGTGCAGATAGTGAACG**-3'** |
| **2** | **Rf-nk_ddPCR-R** | **5'-**CATGTTAAGTTTGCCATAAAATTCG**-3'** |
| **3** | **Rf-nk_ddPCR-probe** | **5'-**/Hex/TGGTGACTT/ZEN/GGGAAGGATGAAGTA/3IABkFQ/**-3'** |
| **4** | **eGFP_ddPCR-F** | **5'-**CAACGAGAAGCGCGATCA**-3'** |
| **5** | **eGFP_ddPCR-R** | **5'-**CGCGATATTACTTGTACAGCTC**-3'** |
| **6** | **eGFP_ddPCR-probe** | **5'-**/56-FAM/CCTGCTGGA/ZEN/GTTCGTGACCGCC/3IABkFQ/**-3'** |
| **7** | ***GCTL-3*_ddPCR-F** | **5'-**CATGGAAGGGAAATTCATATGG**-3'** |
| **8** | ***GCTL-3*_ddPCR-R** | **5'-**GGGTGTATTCTTGGTAGGC**-3'** |
| **9** | ***GCTL-3*_ddPCR-probe** | **5'-**/56FAM/CCGTCAACT/ZEN/ACACCAACTGGGCGT/3IABkFQ/**-3'** |

**Table S4. Primer designs for PCR and sequencing, related to Figure 1 and Figure S1**

| No | Name | Sequence | region |
|---|---|---|---|
| **1** | **mt*GCTL-3*_F1** | **5'-**CGACCATCTTCGATTTGCAGTG**-3'** | **L arm forward** |
| **2** | **mt*GCTL-3*_R1** | **5'-**GTGCAGTCAGCAAAGTGACG**-3'** | **L arm reverse** |
| **3** | **wt*GCTL-3*_F2** | **5'-**ATCGGCGCGGTAGAATACTG**-3'** | **L arm to Pub promoter** |
| **4** | **wt*GCTL-3*_R2** | **5'-**TTAGTCAAAAGCGCAATCGGC**-3'** | **L arm to genome** |
| **5** | **mt*GCTL-3*_F3** | **5'-**CCGACAACCACTACCTGAGC**-3'** | **R arm forward** |
| **6** | **wt*GCTL-3*_R3** | **5'-**CCAATATGCAGGGAAAAAGCAGG**-3'** | **R arm reverse** |
| **7** | **wt*GCTL-3*_F4** | **5'-**ATTCGGGAGAACCGAAAGGA**-3'** | **R arm to genome** |
| **8** | **wt*GCT-L3*_R4** | **5'-**TCTTTATCTGGTGCAAAGTGCT**-3'** | **R arm to eGFP** |

**Table S5. Survival data: Survival probabilities at 12 days after experiment start, related to Figure 3**

| | Median | SE | Lower bound | Upper bound |
|---|---|---|---|---|
| **Control untreated** | 0.979 | 0.0147 | 0.951 | 1 |
| **Control treated** | 0.853 | 0.0364 | 0.784 | 0.927 |
| **Mutant untreated** | 0.937 | 0.0250 | 0.889 | 0.987 |
| **Mutant treated** | 0.905 | 0.0300 | 0.848 | 0.966 |

**Table S6. Primer designs for C-type lectin real-time PCR analysis, related to Figure 1 and Figure S1**

| Gene | Name | sense primer sequence | antisense primer sequence |
|---|---|---|---|
| **AAEL029058** | ***GCTL3*** | ACAGCCCGTCAACTACACCAAC | CCACAACGACTCAGAAAATCAG |
| **AAEL018265** | ***CTL9*** | GGCGGGGAACAATCAAAAGC | CGATTTCCAGTTCAAGCCTCG |
| **AAEL009338** | ***CTL10*** | ATGGAGCTTACGTGGGATTG | GCCGAGTTTTACTGGGATTG |
| **AAEL008299** | ***CTL11*** | AATCTGGTCATGGTGGGTTC | CAGTGGAAGTTTCCCAGCTC |
| **AAEL008681** | ***CTL12*** | TTCGCTGGCATAAACTGTTG | ACTGCATTCTGCGAGATGTG |
| **AAEL012353** | ***CTL15*** | GCACTCATGCTCAATCCAAG | CCTTTACTACGGCGTTGTGC |
| **AAEL005482** | ***CTL18*** | GTACCCCATTCGGACACTTG | TTTCGGGCTGTAAACTGAGG |
| **AAEL011404** | ***CTL19*** | TGGATATTTCGGTGTTTGGCTTGG | AGTTCTCGCCGTATTCGCTAGG |
| **AAEL013853** | ***CTLGA2*** | GCCAACAGAATTATCCACGAGC | CGTCTAGCCAGTCCTTTTCGG |
| **AAEL011070** | ***CTLGA3*** | TCTGCCTAGCCGAACCAAAG | AATAATTGTGTCCACGGTACTGG |
| **AAEL005641** | ***CTLGA5*** | AACATTTTTCCATTGGCACTCA | ACATTCCCTATCGTTCCACTTC |
| **AAEL014382** | ***CTLMA14*** | TCCCCTAAGAAATCAGACGGTG | GTCATTCCCATTCCATTGCAGT |
| **AAEL009496** | ***RPS7*** | CAACAGCAAGAAGGCTATCG | TTGCCGGAGAACTTCTTTTC |

**Table S7. Primer designs for reverse transcription PCR in JAK/STAT, Toll, IMD, autophagy, RNAi, and apoptosis pathways, related to Figure 4.**

| Gene | Name | sense primer sequence | antisense primer sequence |
|---|---|---|---|
| **AAEL012471** | ***Dome*** | AAACGGTGGCAAAATGAACT | CTCCAGACCGGTGAGATTGT |
| **AAEL012553** | ***Hop*** | CCGGACTTTATCGAGCTGTC | ATCTGGTTCACTCCGTCGTC |
| **AAEL007768** | ***MyD88*** | GGCGAGGGTTGTTTCAAGTA | TCCCATCTGTCGATTAAGCC |
| **AAEL010083** | ***IMD*** | TCATTCCGCGAAGGGCTGGC | AGCGCAGAAACATCGTTCGCA |
| **AAEL004522** | ***GAM*** | CGGACCATCAAGCATTTCTCAA | CCAGACGGTGGGTAGAACA |
| **AAEL007696** | ***REL 1*** | GACTCGTCGGAGCTGAAATC | CGGTTTGTTCAGGTTGTTGA |
| **AAEL000709** | ***Cactus*** | TCTTGCGTTGAAGTGAGTGG | GACCCTCTGAAAGGGAAAGG |
| **AAEL006794** | ***Dcr 2*** | CGGGCAAACCCTGTTACATC | TGTTGGATCCTGCGCAAAC |
| **AAEL000200** | ***Vago 1*** | GCATTTGCCGGTCAGAGC | CTCTTCATCGGGATCGAG |
| **AAEL003849** | ***DEFE*** | CCCGAAAGGACCAACCATGA | TTTGCAAAAGGGCGAGCTTC |
| **AAEL003389** | ***Attacin*** | GGACTCCGGCGATTAAGGAG | TCTTCTTGGACCCGAAACGG |
| **AAEL004833** | ***Diptericin A*** | CCAATTCAGGAAGTGGAACC | TGTTGATGGGTAGCTCCAAA |
| **AAEL013441** | ***Toll9A*** | TCAGTCGATGGTGCCAGTTC | CGTGGCCACTTGATGTAGGT |
| **AAEL015099** | ***PIAS*** | GCTGCAACGCATGAAAACTA | CAGACGGGACAGTTCCAAGT |
| **AAEL017251** | ***argonaute-2*** | ACAACAGCAACAATCCCAGA | GTGGACGTTGATCTTGTTGG |
| **AAEL002286** | ***APG 5*** | CCAGGACTTGTTGGAGGACT | GTCCGGATAGCTGAGGTGTT |
| **AAEL014148** | ***dredd*** | GTGGCTGTTATGCGAGAAGA | AGCGTAGTTCTGCCTGAGGT |
| **AAEL001932** | ***FADD*** | GGGACCGTCGAACACTTCTT | CACTCAGCTGCATTAACCGC |
| **AAEL000718** | ***vir-1*** | GCCAAAGTCCGGTATTCTTC | TTCACGAGATCGTCAAGGTAA |
| **AAEL027860** | ***Caspar*** | GAATCCGAGCGAGCCGATGC | CGTAGTCCAGCGTTGTGAGGTC |
| **AAEL005963** | ***Casp-3*** | CGACCCAAAGCAAGGACTCA | CAGCTGCAATCGTCAAACCC |
| **AAEL020559** | ***STAT*** | CACACAAAAAGGACGAAGCA | TCCAGTTCCCCTAAAGCTCA |
| **AAEL019728** | ***SOCS36E*** | CCACTGTTTGGTGCCGGATTTGC | GCGTGCAGCGACCGGTTGTA |
| **AAEL007624** | ***REL 2*** | TACGAGCTCCTTCAACATGC | AGGTCTGCAGTTGACCCTCT |
| **AAEL004223** | ***Cec B*** | GCTGAAGAAGCTGGGAAAAAAG | CTTCCCAGTCCCTTGATGCC |
| **AAEL000611** | ***Cec E*** | CGAAGCCGGTGGTCTGAAG | ACTACGGGAAGTGCTTTCTCA |
| **AAEL015515** | ***Cec G*** | GTTATTTCTCCTGATCGCCG | CTCGTTTTCCTGCACCTCCC |
| **AAEL000621** | ***Cec N*** | CGGCAAGAAATTGGAAAAAGTC | GAATCGATCATCCTAGGGCC |
| **AAEL003841** | ***Def A*** | AACTGCCGGAGGAAACCTAT | AATGCAATGAGCAGCACAAG |
| **AAEL003832** | ***Def C*** | CTTTGTTTGATGAACTTCCGGAG | GAACCCACTCAGCAGATCGC |
| **AAEL003857** | ***Def D*** | GGCGTTGGTGATAGTGCTTG | CACACCTTCTTGGAGTTGCAG |
| **AAEL009496** | ***RPs7*** | GCAGACCACCATTGAACACA | CACGTCCGGTCAGCTTCTTG |

**Table S9. Summary of germline phenotypes. Descriptions of follicles identified in both control and mutant mosquitoes, including phenotype and % of follicles for each type identified in the two genotypes, related to Figure 7**

| **Contents of follicle** | **Phenotype** | **Interpretation** | **% identified in Control (n=393)** | **% identified in *GCTL-3*$^{-/-}$ (n=411)** |
|---|---|---|---|---|
| 7NC*+1oc † | Follicle contains 8 cells | Normal | 93.35 | 50.61 |
| 3NC+1oc | Follicle contains 4 cells | Follicle with clear 4 or 3+1 cells | 3.33 | 3.41 |
| 15NC+1oc | Follicle contains 16 cells | Includes 15+1 follicle | 0.95 | 17.27 |
| Defect in encapsulation | Including fused follicle | Includes all abnormal nurse cells (not 3+1, 4, 7+1, 8, 15+1 and 16 follicles) | 0.95 | 22.87 |
| Defect in oocyte specification | Follicle contains only nurse cells | Includes 4, 8 and 16 NC (no oocyte specification) | 0.24 | 6.57 |
| Without germ cells | Agametic germarium | Difficult to count in mutant line | 1.19 | NA |

*NC: nurse cells., †oc: oocyte

**Table S10. Primer designs for dsRNA, related to Figure 8.**

| Gene | Name | primer sequence |
|---|---|---|
| **AAEL003389** | ***Attacin*-F** | TAATACGACTCACTATAGGGCCGGAATTTTCGGTTCC CAC |
| **AAEL003389** | ***Attacin*-R** | TAATACGACTCACTATAGGGCCGGTTGAGTTCGGCTT TTG |
| **AAEL004522** | ***Gambicin*-F** | TAATACGACTCACTATAGGGTAAGAAGCTGCAGTGAC TGTCAGAAGCGGT |
| **AAEL004522** | ***Gambicin*-R** | TAATACGACTCACTATAGGGTTCTTCAATATCAATCAAT GACACACATGCCC |
| **pUC19 DNA** | **LacZ-F** | TAATACGACTCACTATAGGGTGACCATGATTACGCCAA GC |
| **pUC19 DNA** | **LacZ-R** | TAATACGACTCACTATAGGGATGCGGCATCAGAGCAG ATT |

**Table S11. Results of two-way ANOVA on dsRNA experiment. Significant differences between groups are bolded, related to Figure 8.**

**Egg laying**

| Group | Difference | lwr | upr | Adj P val |
|---|---|---|---|---|
| GCTL:ATT-Control:ATT | -12.09578544 | -27.416993 | 3.2254225 | 0.2409433 |
| Control:Cont-Control:ATT | -4.28544061 | -17.066909 | 8.4960281 | 0.9708499 |
| GCTL:Cont-Control:ATT | -62.26624738 | -75.308459 | -49.2240356 | **0.0000000** |
| Control:dsLac-Control:ATT | -2.29960317 | -15.180403 | 10.5811963 | 0.9993986 |
| GCTL:dsLac-Control:ATT | -60.02842377 | -73.749344 | -46.3075031 | **0.0000000** |
| Control:GAM-Control:ATT | -0.02525253 | -13.666209 | 13.6157035 | 1.0000000 |
| GCTL:GAM-Control:ATT | -14.96031746 | -30.447006 | 0.5263709 | 0.0668511 |
| Control:Cont-GCTL:ATT | 7.81034483 | -6.822509 | 22.4431986 | 0.7332766 |
| GCTL:Cont-GCTL:ATT | -50.17046194 | -65.031611 | -35.3093127 | **0.0000000** |
| Control:dsLac-GCTL:ATT | 9.79618227 | -4.923514 | 24.5158787 | 0.4634808 |
| GCTL:dsLac-GCTL:ATT | -47.93263833 | -63.392850 | -32.4724271 | **0.0000000** |
| Control:GAM-GCTL:ATT | 12.07053292 | -3.318754 | 27.4598198 | 0.2485990 |
| GCTL:GAM-GCTL:ATT | -2.86453202 | -19.911291 | 14.1822267 | 0.9995965 |
| GCTL:Cont-Control:Cont | -57.98080677 | -70.207020 | -45.7545937 | **0.0000000** |
| Control:dsLac-Control:Cont | 1.98583744 | -10.068041 | 14.0397161 | 0.9996461 |
| GCTL:dsLac-Control:Cont | -55.74298316 | -68.690750 | -42.7952165 | **0.0000000** |
| Control:GAM-Control:Cont | 4.26018809 | -8.602808 | 17.1231846 | 0.9727771 |
| GCTL:GAM-Control:Cont | -10.67487685 | -25.480907 | 4.1311528 | 0.3551060 |
| Control:dsLac-GCTL:Cont | 59.96664420 | 47.636626 | 72.2966620 | **0.0000000** |
| GCTL:dsLac-GCTL:Cont | 2.23782361 | -10.967403 | 15.4430501 | 0.9995734 |
| Control:GAM-GCTL:Cont | 62.24099485 | 49.118875 | 75.3631146 | **0.0000000** |
| GCTL:GAM-GCTL:Cont | 47.30592992 | 32.274235 | 62.3376251 | **0.0000000** |
| GCTL:dsLac-Control:dsLac | -57.72882060 | -70.774652 | -44.6829893 | **0.0000000** |
| Control:GAM-Control:dsLac | 2.27435065 | -10.687352 | 15.2360532 | 0.9994632 |
| GCTL:GAM-Control:dsLac | -12.66071429 | -27.552577 | 2.2311481 | 0.1621086 |
| Control:GAM-GCTL:dsLac | 60.00317125 | 46.206273 | 73.8000695 | **0.0000000** |
| GCTL:GAM-GCTL:dsLac | 45.06810631 | 29.443887 | 60.6923258 | **0.0000000** |
| GCTL:GAM-Control:GAM | -14.93506494 | -30.489108 | 0.6189781 | 0.0701576 |

**Larvae hatching**

| Group | Difference | lwr | upr | Adj P val |
|---|---|---|---|---|
| GCTL:ATT-Control:ATT | -20.985441 | -33.948291 | -8.022590 | **0.0000337** |
| Control:Cont-Control:ATT | -2.450958 | -13.265005 | 8.363089 | 0.9971965 |
| GCTL:Cont-Control:ATT | -68.560587 | -79.595242 | -57.525932 | **0.0000000** |

| | | | | |
|---|---|---|---|---|
| Control:dsLac-Control:ATT | -1.267460 | -12.165549 | 9.630628 | 0.9999661 |
| GCTL:dsLac-Control:ATT | -65.437726 | -77.046618 | -53.828835 | **0.0000000** |
| Control:GAM-Control:ATT | -5.702525 | -17.243761 | 5.838710 | 0.8035555 |
| GCTL:GAM-Control:ATT | -22.053175 | -35.156033 | -8.950316 | **0.0000131** |
| Control:Cont-GCTL:ATT | 18.534483 | 6.154030 | 30.914936 | **0.0001855** |
| GCTL:Cont-GCTL:ATT | -47.575146 | -60.148754 | -35.001539 | **0.0000000** |
| Control:dsLac-GCTL:ATT | 19.717980 | 7.264052 | 32.171908 | **0.0000561** |
| GCTL:dsLac-GCTL:ATT | -44.452285 | -57.532743 | -31.371828 | **0.0000000** |
| Control:GAM-GCTL:ATT | 15.282915 | 2.262465 | 28.303365 | **0.0092846** |
| GCTL:GAM-GCTL:ATT | -1.067734 | -15.490525 | 13.355057 | 0.9999985 |
| GCTL:Cont-Control:Cont | -66.109629 | -76.453890 | -55.765368 | **0.0000000** |
| Control:dsLac-Control:Cont | 1.183498 | -9.014956 | 11.381951 | 0.9999666 |
| GCTL:dsLac-Control:Cont | -62.986768 | -73.941516 | -52.032021 | **0.0000000** |
| Control:GAM-Control:Cont | -3.251567 | -14.134593 | 7.631458 | 0.9848960 |
| GCTL:GAM-Control:Cont | -19.602217 | -32.129189 | -7.075245 | **0.0000729** |
| Control:dsLac-GCTL:Cont | 67.293127 | 56.861040 | 77.725214 | **0.0000000** |
| GCTL:dsLac-GCTL:Cont | 3.122861 | -8.049716 | 14.295438 | 0.9898116 |
| Control:GAM-GCTL:Cont | 62.858062 | 51.755799 | 73.960324 | **0.0000000** |
| GCTL:GAM-GCTL:Cont | 46.507412 | 33.789511 | 59.225314 | **0.0000000** |
| GCTL:dsLac-Control:dsLac | -64.170266 | -75.207983 | -53.132549 | **0.0000000** |
| Control:GAM-Control:dsLac | -4.435065 | -15.401603 | 6.531473 | 0.9215275 |
| GCTL:GAM-Control:dsLac | -20.785714 | -33.385307 | -8.186121 | **0.0000215** |
| Control:GAM-GCTL:dsLac | 59.735201 | 48.062027 | 71.408375 | **0.0000000** |
| GCTL:GAM-GCTL:dsLac | 43.384551 | 30.165331 | 56.603772 | **0.0000000** |
| GCTL:GAM-Control:GAM | -16.350649 | -29.510495 | -3.190804 | **0.0043802** |

## Fig. S1

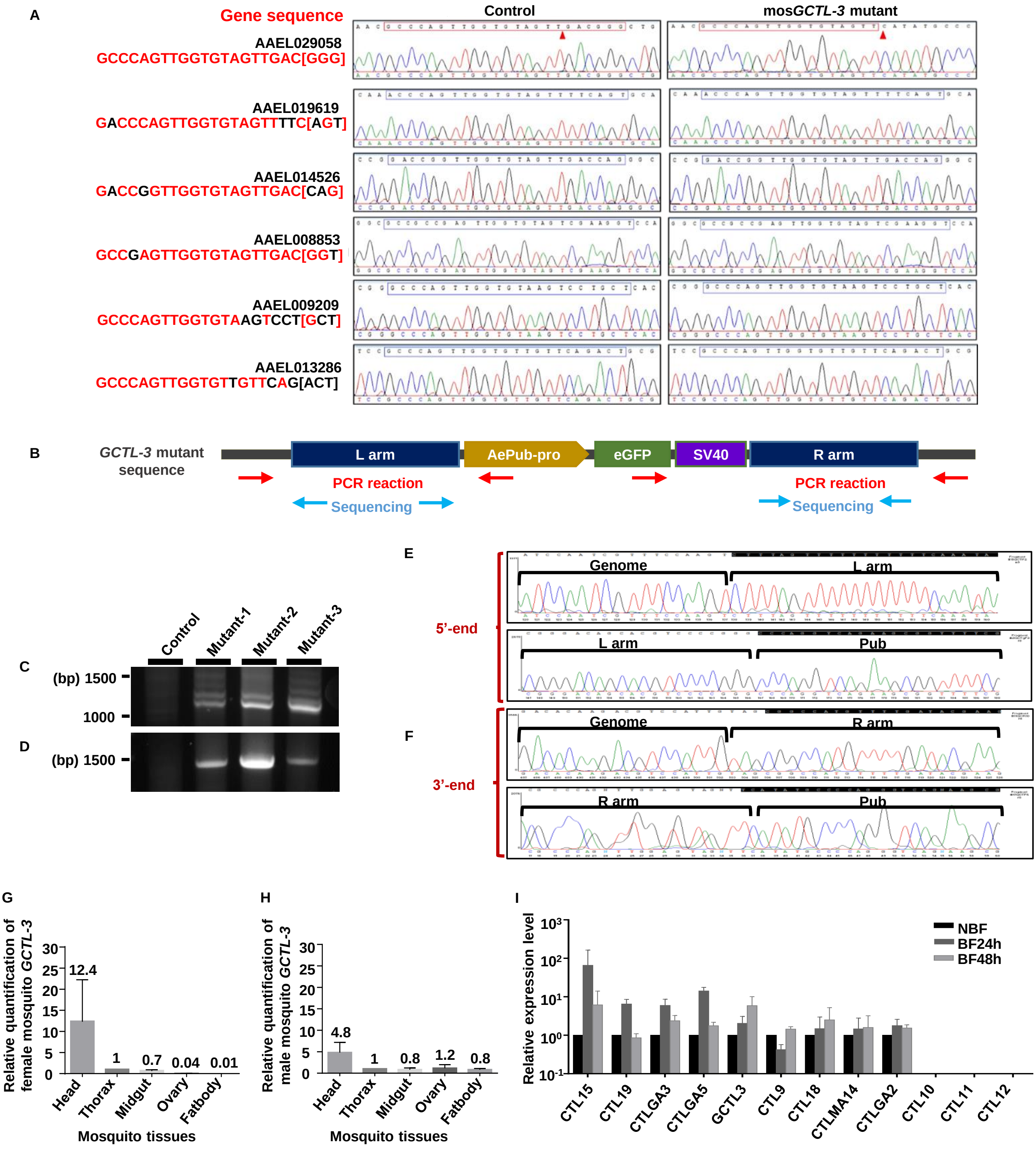
A
Gene sequence
Control
mosGCTL-3 mutant
AAEL029058
GCCCAGTTGGTGTAGTTGAC[GGG]
AAEL019619
GACCCAGTTGGTGTAGTTTTC[AGT]
AAEL014526
GACCGGTTGGTGTAGTTGAC[CAG]
AAEL008853
GCCGAGTTGGTGTAGTTGAC[GGT]
AAEL009209
GCCCAGTTGGTGTAAGTCCT[GCT]
AAEL013286
GCCCAGTTGGTGTTGTTCAG[ACT]
B
GCTL-3 mutant sequence
L arm
AePub-pro
eGFP
SV40
R arm
PCR reaction
Sequencing
PCR reaction
Sequencing
C
Control
Mutant-1
Mutant-2
Mutant-3
(bp) 1500
1000
D
(bp) 1500
E
5'-end
Genome
L arm
L arm
Pub
F
3'-end
Genome
R arm
R arm
Pub
G
Relative quantification of female mosquito GCTL-3
12.4
1
0.7
0.04
0.01
Head
Thorax
Midgut
Ovary
Fatbody
Mosquito tissues
H
Relative quantification of male mosquito GCTL-3
4.8
1
0.8
1.2
0.8
Head
Thorax
Midgut
Ovary
Fatbody
Mosquito tissues
I
Relative expression level
NBF
BF24h
BF48h
CTL15
CTL19
CTLGA3
CTLGA5
GCTL3
CTL9
CTL18
CTLMA14
CTLGA2
CTL10
CTL11
CTL12

**Fig. S1. *Aedes GCTL-3* gene locus knock-out by CRISPR/Cas9, related to Figure 1, Table S1 and Table S4.** (A) sequencing analyses of the five potential target sites identified for *GCTL-3* knock-out. (B) Primer sets for PCR reaction and sequencing confirmation (red and blue arrows in respectively) of break point detection. (C to D) Gel results of (C) 5'-end and (D) 3'-end break points of genomic DNA in three different *GCTL-3*$^{-/-}$ mosquito lines and detected by PCR. (E to F) Sequencing results of (E) 5'-end and (F) 3'-end break points in *GCTL-3*$^{-/-}$ mosquitoes found three *GCTL-3* mutants having an eGFP marker inserted correctly into the target site. (G) Relative quantification of mos*GCTL-3* by real time PCR in control female (N=10) and (H) male (N=10) mosquitoes as compared to whole body expression levels. Females have higher expression levels in the thorax and midgut but lower levels in the ovary and fatbody. *GCTL-3* expression level in the head was higher in females than males. Data was collected across three biological repeats; data are represented as mean ± SD. (I) Expression levels of lectins in female *A. aegypti* midgut. Data are represented as mean ± SD.

Fig. S2

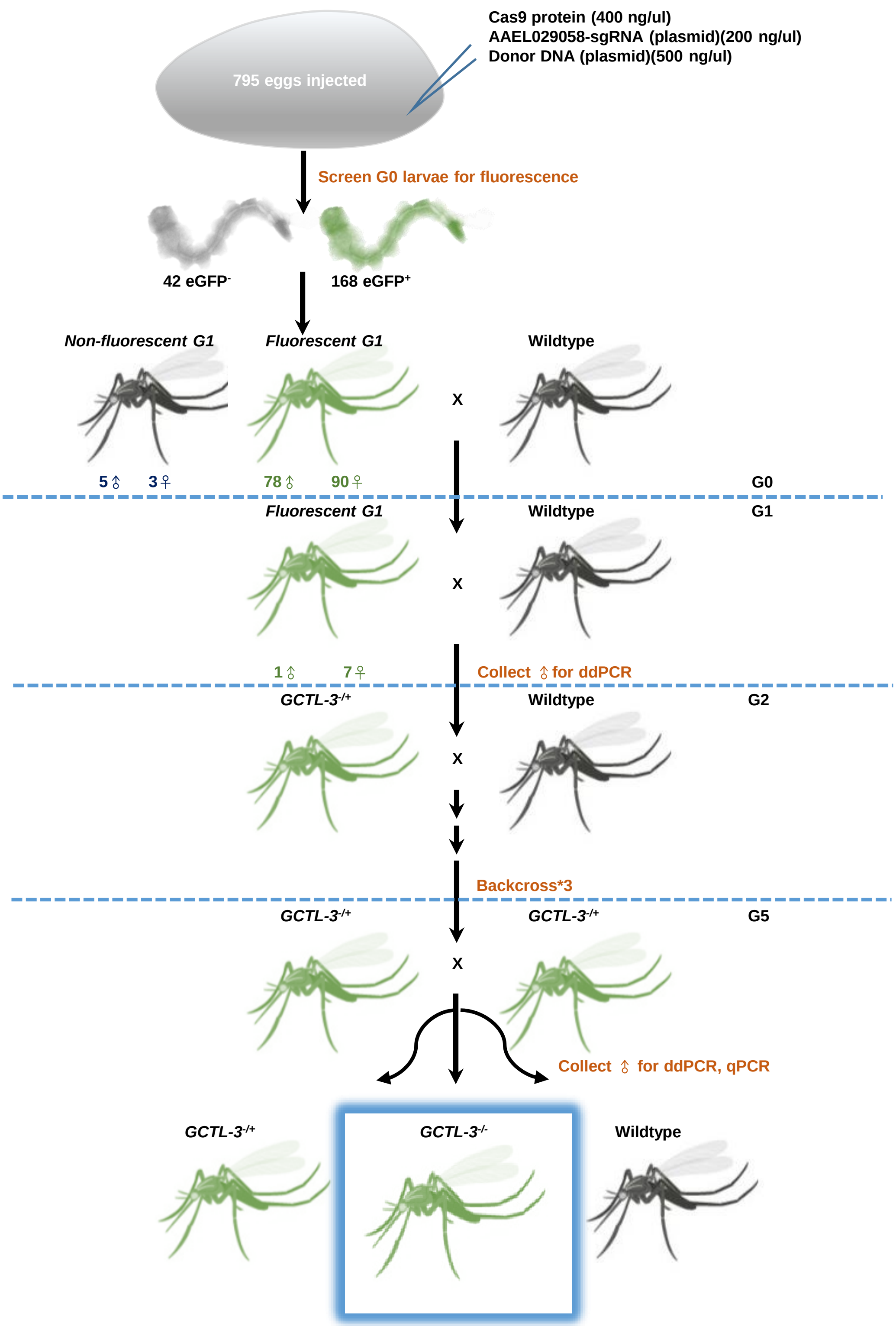


**Fig. S2. Schematic of injection and screening strategies to establish an insertion of an eGFP homozygous mutant (green), related to Figure 1 and Table S2**

# Fig. S3

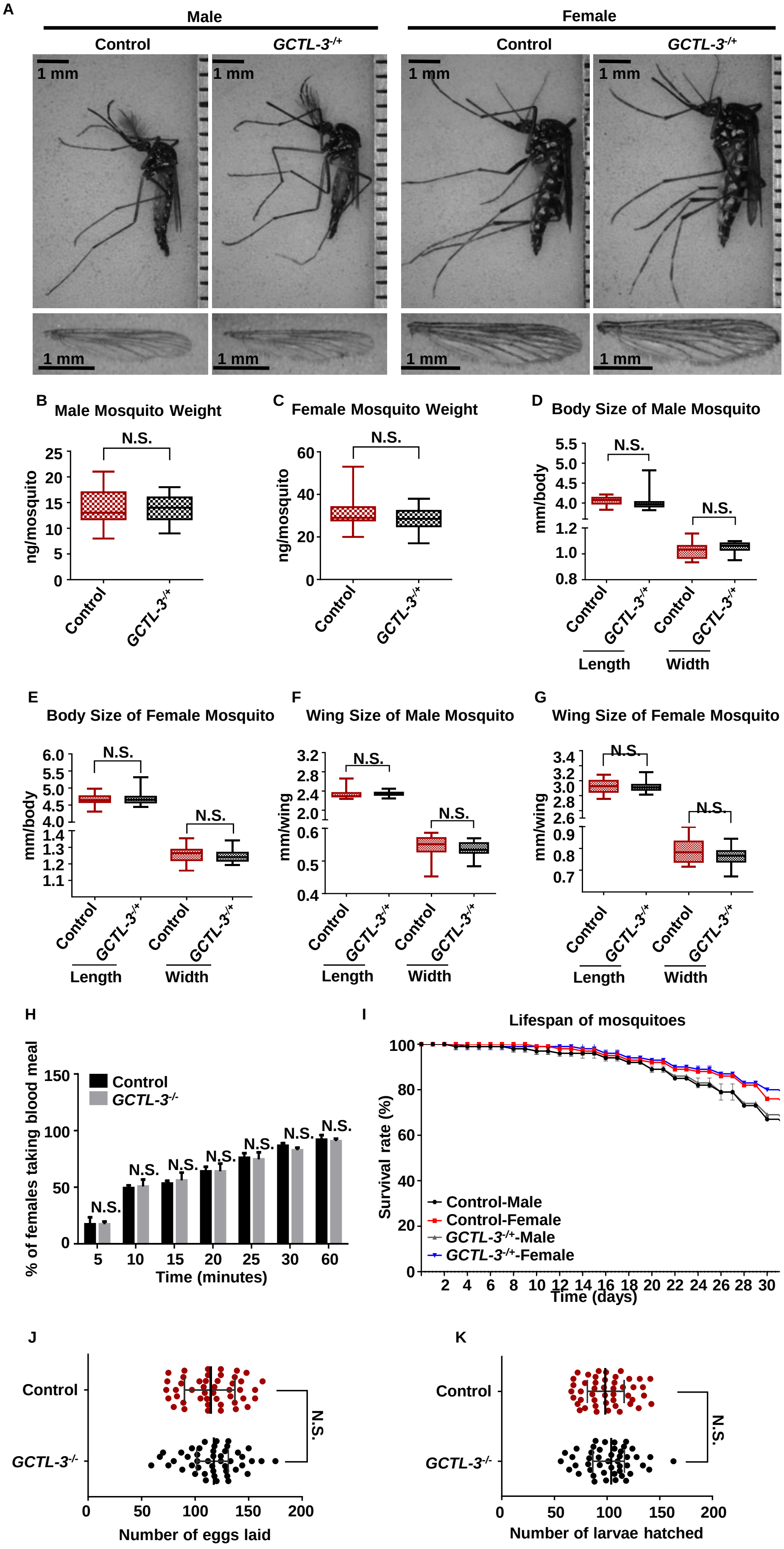
A
Male
Female
Control
GCTL-3-/+
1 mm
B Male Mosquito Weight
ng/mosquito
N.S.
C Female Mosquito Weight
D Body Size of Male Mosquito
mm/body
Length
Width
E Body Size of Female Mosquito
F Wing Size of Male Mosquito
mm/wing
G Wing Size of Female Mosquito
H
% of females taking blood meal
GCTL-3-/-
Time (minutes)
I Lifespan of mosquitoes
Survival rate (%)
Control-Male
Control-Female
GCTL-3-/+-Male
GCTL-3-/+-Female
Time (days)
J
Number of eggs laid
K
Number of larvae hatched

**Fig. S3. *GCTL-3$^{-/+}$* mosquitoes show no change in physiology and lifespan compared to controls, related to Figure 1.** (A) Phenotypes of control and *GCTL-3$^{-/+}$* mosquitoes of both sexes. (B–G) Quantification of (B and C) mosquito weight (Sample sizes: Control/mutant females=30/30; Control/mutant males=30/30); data are represented as mean ± SD. (D and E) body size (Sample sizes: Control/mutant females=23/22; Control/mutant males=18/18) and (F and G) wing size (Sample sizes: Control/mutant females=23/23; Control/mutant males=21/21). Data are represented as mean ± SD. (H) Percentage of female control and mutant mosquitoes (N=25 for each group) identified by eye as taking a blood meal at set intervals after being provided with a blood source. All samples were taken from the same generation. Three independent experiments were conducted. Data are represented as mean ± SD. Mann-Whitney tests were used to test for potential significant differences between groups (I) Lifespans of mosGCTL-3 heterozygous mutants and control mosquitoes (N=100, two independent experiments). Data are represented as mean ± SD. (J) Number of eggs and (K) number of hatched larvae for *GCTL-3$^{-/+}$* mutants and controls. Sample sizes: control=46; *GCTL-3$^{-/+}$*=46. Mann-Whitney tests were used to test for potential significant differences between groups. Data are represented as mean ± SD.

**Fig. S4**

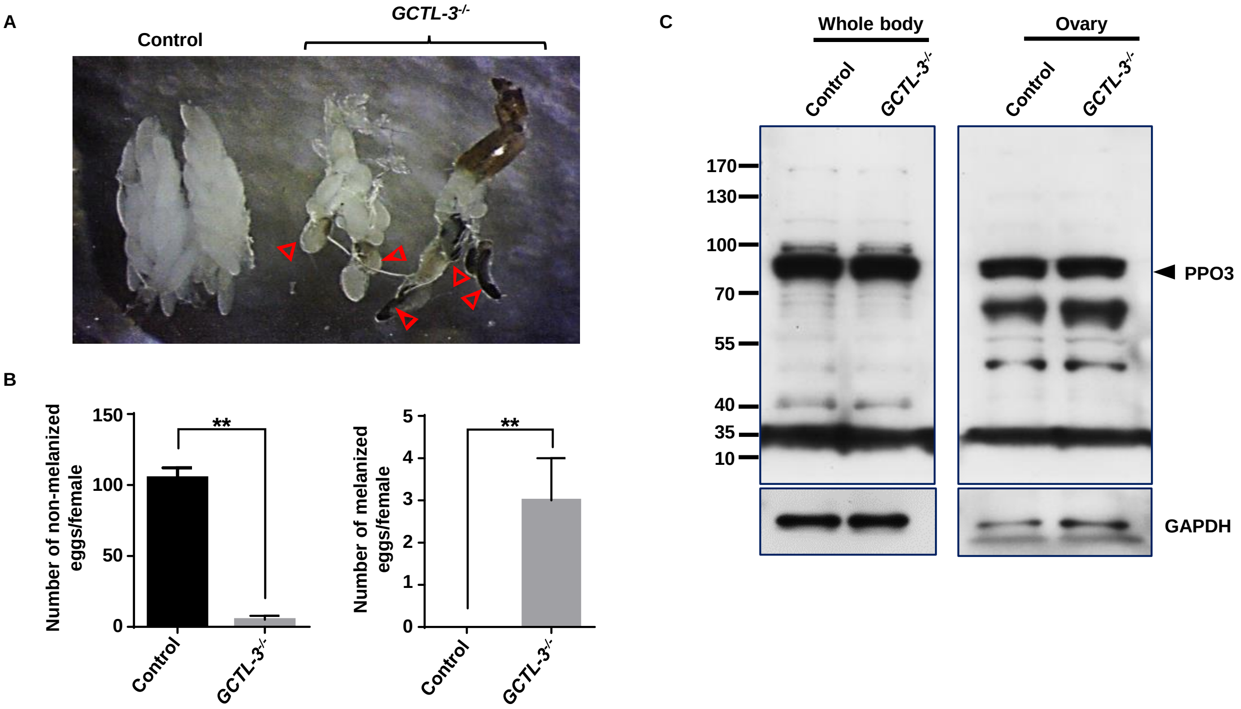


**Fig. S4. Melanization in *GCTL3*$^{-/-}$ ovaries, related to Figure 5**. (A) Phenotype of embryos in control (left) and *GCTL-3*$^{-/-}$ mosquitoes (middle and right). (B) Quantification of differences in egg types/counts between control and mutant mosquitoes; *GCTL-3*$^{-/-}$ mutants show significant reductions in overall egg counts (N=2) per female but have significantly greater numbers of melanized eggs (N=3) compared to controls (Student t-test; $p$=0.0029 and $p$=0.0065). Data are represented as mean ± SD. (C) PPO3 expression level in mutant and control mosquito whole bodies as well as ovaries as detected by western blotting. 40ug protein sample was used. PPO3 was the primary antibody diluted to 1:5000 in PBST containing 2% BSA; anti-rabbit was used as the secondary antibody diluted to 1:13000. GAPDH (1:10000) was used as a negative control, with anti-mouse (1:10000) the second antibody.

Fig. S5

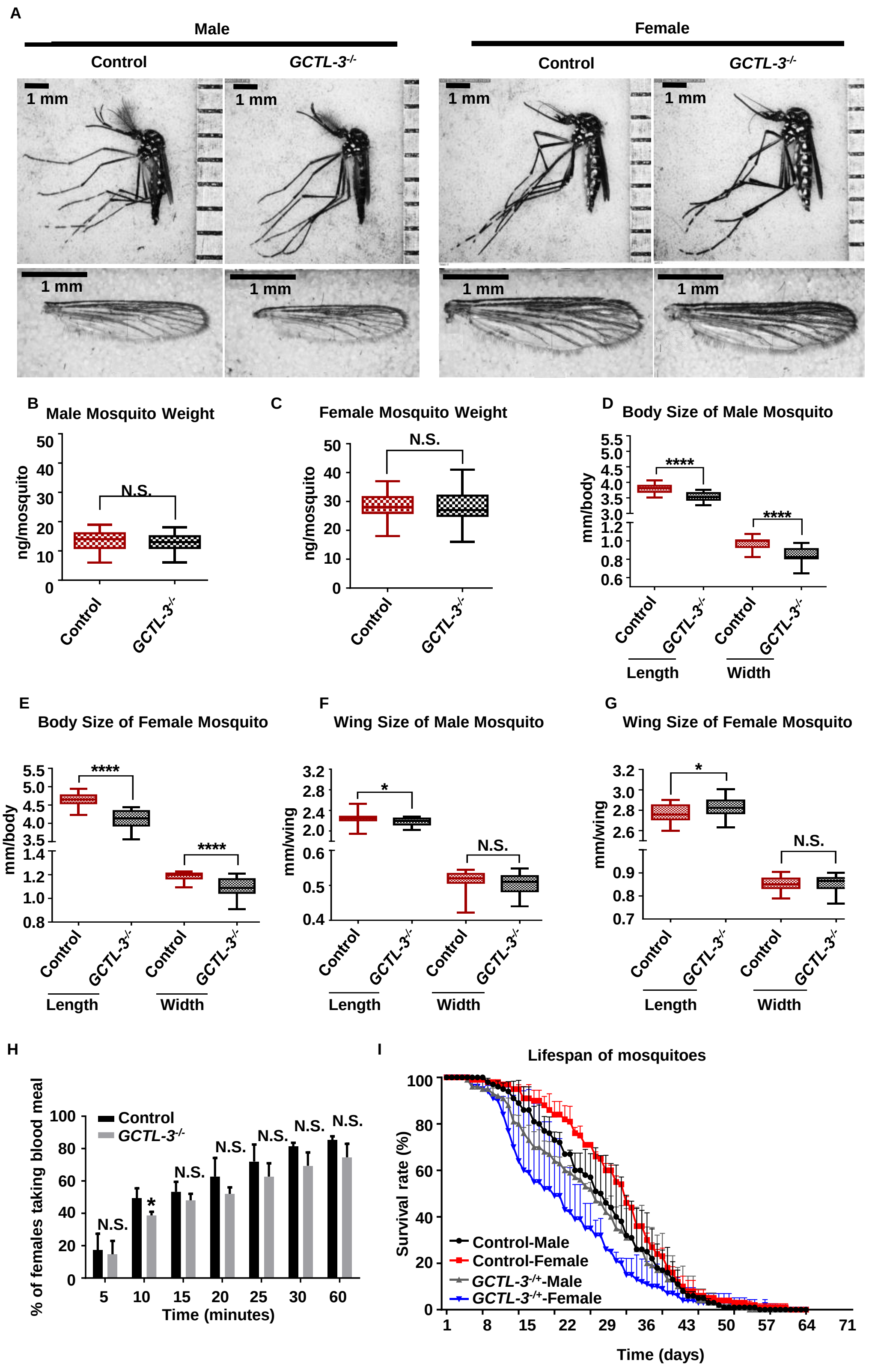
A
Male
Female
Control
GCTL-3-/-
1 mm
B Male Mosquito Weight
ng/mosquito
N.S.
C Female Mosquito Weight
D Body Size of Male Mosquito
mm/body
****
Length
Width
E Body Size of Female Mosquito
F Wing Size of Male Mosquito
mm/wing
*
G Wing Size of Female Mosquito
H
% of females taking blood meal
Time (minutes)
I Lifespan of mosquitoes
Survival rate (%)
Time (days)
Control-Male
Control-Female
GCTL-3-/+-Male
GCTL-3-/+-Female

**Fig. S5. *GCTL-3$^{-/-}$* mosquitoes show change in physiology and lifespan compared to controls, related to Figure 5.** (A) Phenotypes of control and *GCTL-3$^{-/-}$* mosquitoes of both sexes. (B - G) Quantification of (B and C) mosquito weight (Sample sizes: Control/mutant females=25/25; Control/mutant males=30/30); data are represented as mean ± SD. (D and E) body size (Sample sizes: Control/mutant females=20/22; Control/mutant males=21/21) and (F and G) wing size (Sample sizes: Control/mutant females=21/23; Control/mutant males=22/21). Data are represented as mean ± SD. (H) Percentage of female control and mutant mosquitoes (total number=75 for each group) identified by eye as taking a blood meal at set intervals after being provided with a blood source. All samples taken from the same generation Three independent experiments were conducted. Data are represented as mean ± SD. Single asterisks represent a significant difference determined by the student t-test at $p$<0.05. (I) Lifespans of *mosGCTL-3* homozygous mutants and control mosquitoes (N=100, two independent experiments). Data are represented as mean ± SD.

Fig. S6

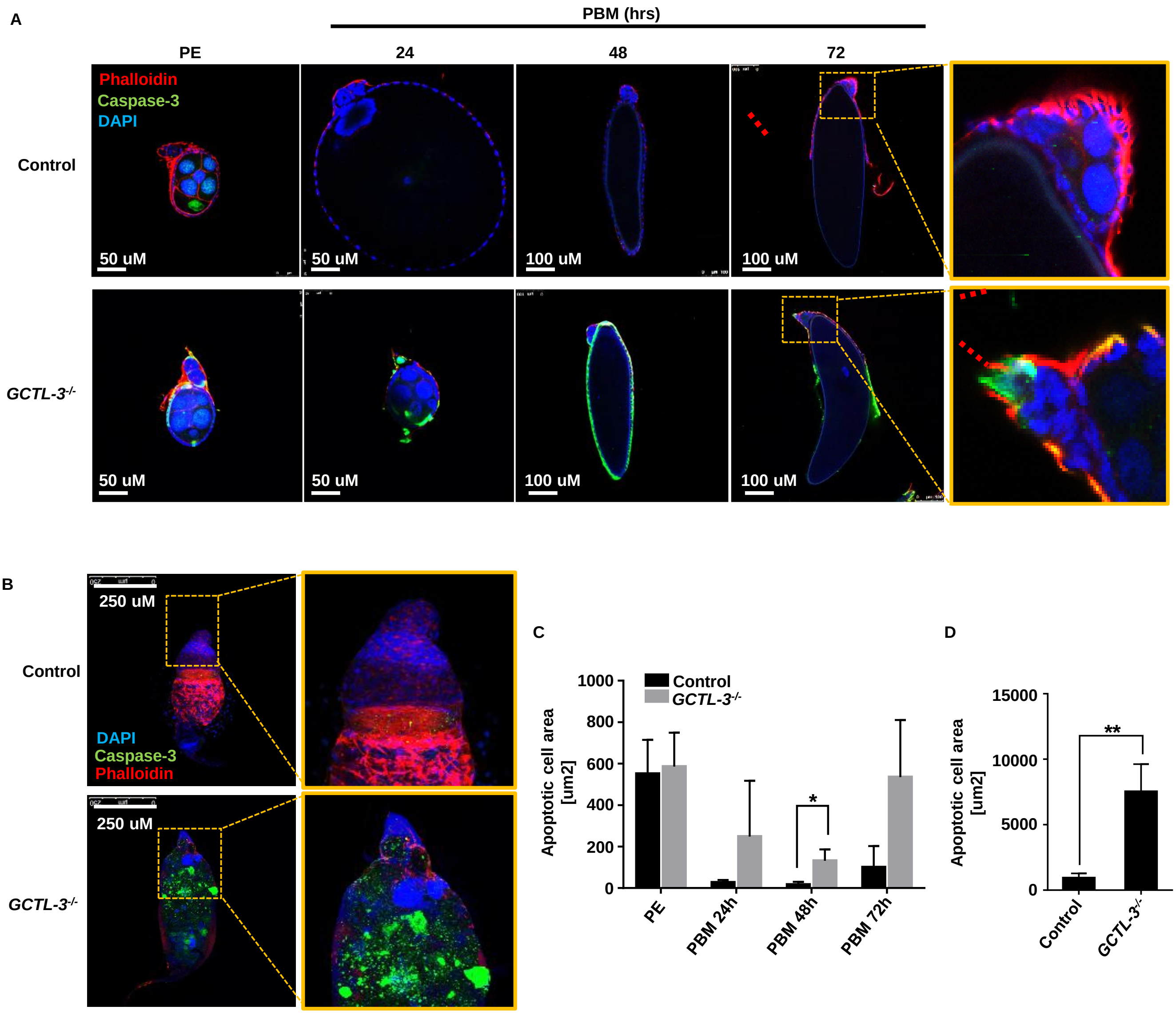


**Fig. S6. Caspase-3 expression levels increased in *GCTL-3*$^{-/-}$ mutant mosquito testes and ovaries post blood meal, related to Figure 6.** (A and C) 72 hours post blood meal, caspase-3 signal was increased in germ line cells of *GCTL-3*$^{-/-}$ ovaries and (B and D) testes. Data are represented as mean ± SD. Cleaved-Caspase-3 used as a primary antibody (1:500 dilution); Alexa Fluor 488 dye as a secondary antibody (1:500) as well as DAPA (1:1000) and Phalloidin (1:500) staining on the cell nucleus and cytoskeletons.

## Fig. S7

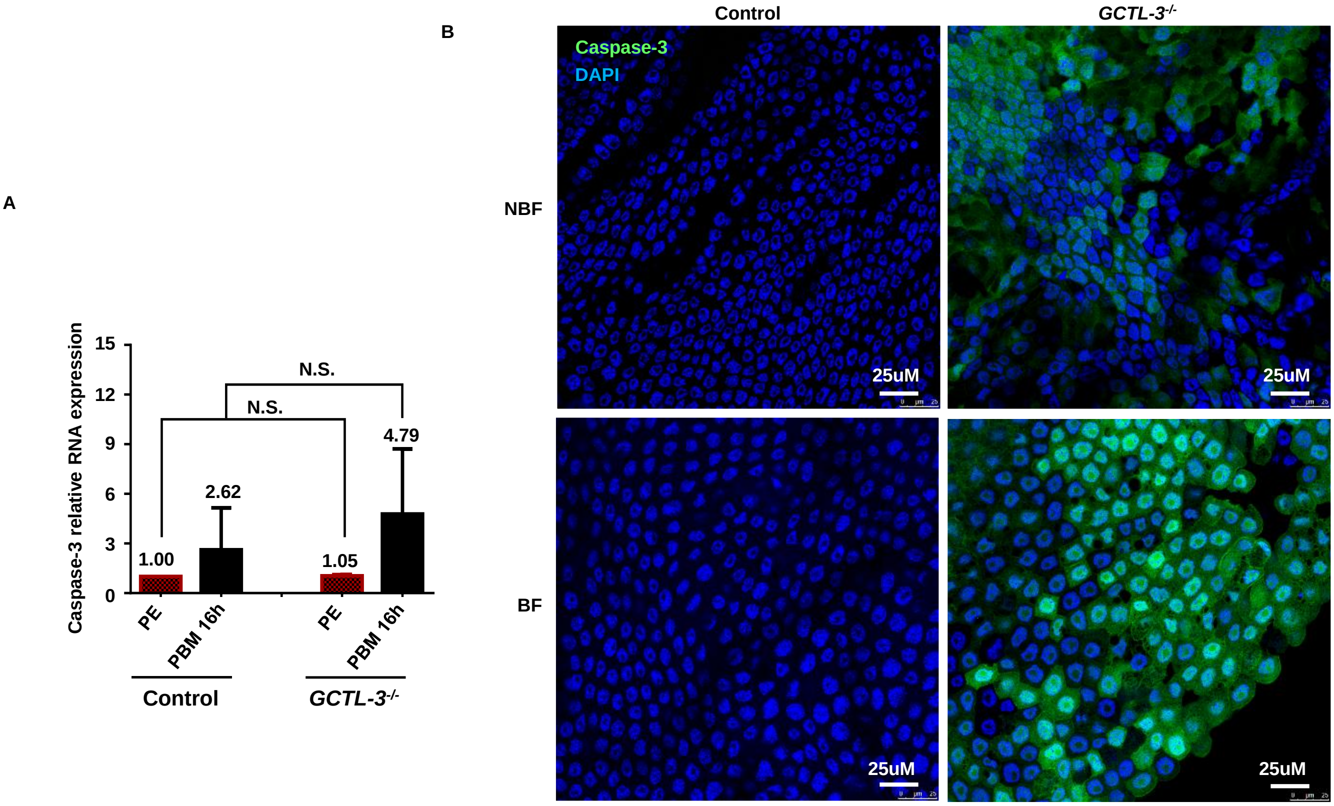


**Fig. S7, see also Table S7. Cleavage Caspase-3 expression in *GCTL-3*$^{-/-}$ midguts, related to Figure 7.** (A) 16 hours post blood meal, midguts of 5 day old *GCTL-3*$^{-/-}$ mosquitoes seemed to show a greater relative increase in caspase-3 RNA expression level than controls (N=10/each group), though no statistically significant difference was found (Mann-Whitney test; $p$=0.3333). Data are represented as mean ± SD. (B) Caspace-3 protein expression levels also appeared higher in mutant midguts than in controls, as checked via immunostaining. Primary antibody (cleave-caspase-3) was used in a 1:500 dilutions whilst second antibody (Alexa-488) was used in 1:500 dilution mixed with 1:1000 DAPI. NFB= non-blood fed; BF= blood fed.

## Transparent Methods

### Plasmid assembly

For the sgRNA and homologous recombination (HR) donor vector, an AaeU6 (AAEL017763) and PUb promoter were generated from *Aedes aegypti* Higgs strain genomic DNA. Detailed construction information is provided in the SI Appendix. A specific sgRNA targeting *GCTL-3* for CRISPR/Cas9 recognition was identified by copying the sequence into the flyCRISPR target finder web application (https://flycrispr.org/). An In-Fusion HD Cloning Kit (Takara Bio USA, Inc.) was used to generate the donor vector.

### Mosquito rearing

All experiments used either the *Aedes aegypti* Higgs strain or mutants generated from this line. Mosquito larvae were reared at 28 °C and fed with a mixture of yeast powder (Taiwan Sugar Corporation) and goose liver powder (#7573, NTN) in a 1:1 ratio. Adults were maintained in a temperature and humidity controlled room (28 °C and ~70% RH) with a 12 hour light/dark cycle and provided with a constant 10% sucrose solution (Das et al., 2007).

### Generation of mutant mosquitoes

Three days after being provided with a blood meal, female Higgs mosquitoes were allowed to lay eggs for 45 minutes. The DNA mixture used for injections was centrifuged at 13,000 rpm for 10 minutes at 4 °C. Then, 1 μL of the mixture was loaded into a glass needle (aluminosilicate tubing with filament, Sutter Instruments, AF100-64-10). The DNA mix contained 200 ng/μL sgRNA, 200 ng/μL Cas9 protein (Invitrogen, B25640), 500 ng/μL HR plasmid and 1x injection buffer (2 mM KCl, 0.1 mM sodium phosphate, pH 6.8) (Kistler et al., 2015; Kyrou et al., 2018; Nijole Jasinskiene, 2007). The tip of the glass needle was broken so that the DNA mix could be ejected. Approximately 20–60 embryos were aligned on wet filter paper and dehydrated before being transferred to a cover slide. About 200~500 μL Halocarbon oil 700 (Sigma, H8898) was used to cover all embryos. An injection needle was used to penetrate the posterior side of each embryo sequentially by moving the microscope plate laterally; care was taken that insertions did not exceed one-tenth the length of the embryonic body. Injection volume was maximally 20 pL.

Post-injection, embryos were transferred to fresh wet filter paper to remove any remaining Halocarbon oil. Injected embryos were kept on this filter paper for four days before hatching. Male and female pupae were sexed prior to adult emergence to obtain male and female virgins. Each surviving injected generation 0 (G0) male adult was outcrossed with three control females. G0 females were pooled together and crossed with control males at a male/female ratio of 1:3 (Lobo et al., 2006). Expression of *eGFP* fluorescence driven by the PUb promoter throughout the whole body of G1 *GCTL-3*$^{-/-}$ mutant mosquitoes was confirmed via use of a stereoscopic

microscope (SZX10, Olympus). Embryo survival rate following injection was 26% (N=210/795) and the success rate for generating *GCTL-3*$^{-/+}$ mutants was 1.1% (SI Appendix, Table S2).

**Plasmid assembly**

For the pBFv-AaeU6_*GCTL-3*-sgRNA vector, a PCR-amplified 520bp AaeU6 promoter w as generated from the AAEL017763 gene locus of *A. aegypti* genomic DNA and then f used with a sgRNA backbone sequence to obtain an AaeU6-sgRNA DNA fragment via primer extension. This AeU6-sgRNA DNA fragment was cloned into the *Eco*RI/*Not*I sites of pBFv-U6.2 plasmids to create a pBFv-AaeU6-sgRNA backbone vector for the target site single guide RNA (sgRNA) constructs for *A. aegypti* (Kondo and Ueda, 2013). A sg RNA sequence specific for *GCTL-3* for CRISPR/Cas9 recognition was identified by copy ing the sequence into a target finder web application (available at http://tools.flycrispr.molbio.wisc.edu/targetFinder/). 5'-GCCCAGTTGGTGTAGTTGACGGG-3' was identified as a CRISPR/Cas9 target in the *GCTL-3* coding sequence. *GCTL-3*-sgRNA fragments were g enerated by primer annealing using *GCTL-3*-sgRNA-F and *GCTL-3*-sgRNA-R. All primer s used in this study were synthesized by Integrated DNA Technologies (IDT, California, USA). The annealed *GCTL-3*-sgRNA fragments were cloned into the *Bsm*BI sites of the pBFv-AaeU6-sgRNA vector in order to generate the pBFv-AaeU6_*GCTL-3*-sgRNA plas mid. For the pCR2-TOPO-*GCTL-3*-attp-loxp-Pub-eGFP HR donor vector, a 1382bp PUb promoter was created from *Aedes aegypti* genomic DNA via PCR using AePUb-PR-F a nd AePUb-PR-Rand then cloned into the *Avr*II/*Not*I sites of a pCR2-TOPO-attp-loxp-3xp3-eGFP HR vector to obtain the pCR2-TOPO-attp-loxp-PUb-eGFP HR vector (Anderson et al., 2010). Left and right homologous recombination flanking sequences of the *GCTL-3* gene were PCR-amplified from *A. aegypti* Higgs strain genomic DNA by using *GCTL-3*-Up-F, *GCTL-3*-Up-R, *GCTL-3*-Down-F and *GCTL-3*-Down-R (SI Appendix, Table S3).

Two PCR homology arms fragments were cloned into the *Nhe*I/*Xma*I and *Nde*I/*Xho*I sites of the pCR2-TOPO-attp-loxp-Pub-eGFP HR vector using the In-Fusion HD Cloning Kit (Takara Bio USA, Inc.) in order to generate the pCR2-TOPO-*GCTL-3*-attp-loxp-Pub-eGFP HR donor vector.

**Single guide RNA design**

The sgRNA targeting *GCTL-3* for CRISPR/Cas9 recognition was designed using the web tool CRISPR Optimal Target Finder on the flyCRISPR website (https://flycrispr.org/target-finder/) to identify the optimal CRISPR target sites and evaluate their specificity. A 360 bp stretch of the *GCTL-3* coding sequence was obtained from AaegL.3 *A. aegypti* genome of Vectorbase for use as the template for CRISPR target finding. The whole template sequence of *GCTL-3* was pasted into the search window. '*A. aegypti*' was selected as the reference genome for the TagScan genome searching algorithm (Iseli et al., 2007). The parameter was chosen to direct the program to identify either all CRISPR targets, CRISPR targets with 5'G for U6 promoter

driving, or CRISPR targets with 5’GG for T7 promoter driving. All sequences with similarity to CRISPR target queries on both strands of the *GCTL-3* coding sequence were identified with their specificity and location information, and a UCSC Genome Browser (http://genome.ucsc.edu/)(Kent et al., 2002) link for each potential off-target site was created. In order to generate a frameshift mutation as close as possible the ATG site, a specific CRISPR target with zero off-target effects located on the anti-sense strand of *GCTL-3*, 5’-GCCCAGTTGGTGTAGTTGACGGG-3’, was selected and introduced into an AaeU6 promoter driving plasmid for sgRNA construction. The detailed user manual of CRISPR Optimal Target Finder is available at https://flycrispr.org/wp-content/uploads/2019/07/flyCRISPR-Optimal-Target-Finder-Manual-29Jul14.pdf.

**PCR and sequencing**

To confirm the mutant insertion site, Taq DNA polymerase (TA110150, Bernardo Scientific, Taiwan) was used to amplify the target site fragment. The PCR product was sequenced by the DNA Sequencing Core Lab at the NHRI, Taiwan by using an Applied Biosystems® 3730XL DNA Analyzer (ThermoFisher Scientific, California, USA). Construction details for all primers are included in SI Appendix, Table S4.

**Characterization of insertion site by digital droplet PCR**

To verify the precision of the *GCTL-3* gene knock-in and to test for potential off-target effects, a ddPCR platform was used to determine the copy number variant of *eGFP* from the *GCTL-3*-HR donor vector and *GCTL-3* alleles. Here, 20 ng of genomic DNA from a single mutant G1 or control male *Aedes aegypti* Higgs strain mosquito was used as the template for ddPCR analysis. The probe and primer sets for *eGFP* and *GCTL-3* were designed within the *eGFP* ORF and straddled the sgRNA of *GCTL-3*. AAEL006597, a known single-copy autosomal gene, was used as a reference, with the reference copy number of AAEL006597 set as two for diploid alleles (Hall et al., 2015). All of the experimental reagents and steps followed the protocol established in the ddPCR Copy Number Variation Assays Product Insert, Ver C (Bulletin #10033173) of Bio-Rad Laboratories (Mazaika and Homsy, 2014). The sequences of all primer/probe sets used in this study are included in SI Appendix, Table S5.

**C-type lectin expression analysis**

Adult female *A. aegypti* Higgs strain mosquitoes were fed mice blood via an artificial membrane for 30 minutes. Successfully blood fed mosquitoes were then maintained in a separate container. Midguts of these mosquitoes (N=23/group) were collected at either day one or day three post-blood meal and the total RNA was extracted using TRI reagent (Merck) following the manufacturer’s protocol. cDNA was reverse transcribed from 2 µg total RNA using SuperScript III Reverse Transcriptase (ThermoFisher Scientific, California, USA) immediately after the total

RNA was extracted.

cDNA from 10 ng total RNA was used as a sample for the relative real-time PCR analysis of CTL expression. Real-time PCRs were performed using a KAPA SYBR FAST ROX Low Kit (KAPA Biosystems) on a ViiA 7 Real-Time PCR system (Thermo Fisher Scientific, California, USA). Three biological replicates were completed, and data were normalized to *A. aegypti* S7 ribosomal protein levels (RPS7; AAEL009496). Primers are listed in SI Appendix, Table S6.

**DENV/ ZIKV infection of mosquitoes and virus titer determination**

For oral infection, $1\times10^7$ PFU/mL virus stock was 1:1 mixed with mouse blood and fed to mosquitoes at 37 °C for 30 minutes via metal plate. For thoracic infection, 400 PFU virus stock was diluted with serum free medium and thoracic into adult female mosquito thorax. After seven days, a whole mosquito was collected for detecting virus titer. To determine the virus titer, $2\times10^5$ BHK or Vero cells were seeded into a 6-well plate and a mosquito was ground and diluted with serum-free DMEM medium. Two hours following infection, the unbound viral particles were removed and 3 mL DMEM medium containing 1% Seaplaque agarose (FMC BioProducts, Rockland, ME, US) and 2% FBS (Gibco, Paisley, UK) was added. After six days of incubation, cells were fixed and stained with 0.5 μL cell staining solution (0.5% Crystal Violet, 1.85% formaldehyde, 50% ethanol, 0.85% NaCl) and then washed with $H_2O$. Plaque numbers were counted and viral titer was determined as plaque forming units per mosquito.

**Host seeking behavior assay**

Five-to-seven day old female Higgs strain or mutant mosquitoes (N=25) were starved for 16 hours and then divided into control and experimental groups, which were kept overnight in a 15×15×15 cm cage under normal rearing conditions. A BALB/c female mouse was placed into each cage at the same time and the number of blood-fed mosquitoes (as determined by eye) was recorded every 5 minutes to 30 as well as 60 minutes. Three independent replicates were conducted.

**Mosquito physiological measurements; body weight, body length, and wing length**

The mosquitoes were anesthetized on ice for five minutes. Mosquitoes were transferred into 1.5 mL Eppendorf tubes after the tubes alone were weighed. A microbalance was used to weigh 25~30 mosquitoes for each group. Mosquito bodies and wings were imaged using a Dino-Lite Digital Microscope.

**16S amplicon sequencing**

Prior to sample collection, 5- to 7- day old female mosquitoes (N=15) were anesthetized via ice sedation for five minutes before being transferred into 1.5 mL Eppendorf tubes containing 70% ethanol for two minutes. After four washes with 1×PBS, mosquito midguts were dissected and

collected into new 1.5 mL Eppendorf tubes containing 1 mL 1×PBS within 15 minutes and stored at -20 °C. Collected samples were delivered to Tools Inc., Taiwan for DNA extraction and 16S Amplicon Sequencing.

**RNA extraction and reverse transcription polymerase chain reaction (RT-PCR)**

Ten non-blood fed and blood fed female Higgs strain and mutant mosquito midguts and fat-bodies were dissected in 1×PBS at room temperature. Total RNA was extracted using TRI Reagent (Sigma-Aldrich; T9424). From each sample, 1 μg total RNA was used for reverse transcription via the SuperScript™ IV Reverse Transcriptase Kit (18090010, ThermoFisher Scientific, California, USA) with random primers. RNase-free water was used to dilute cDNA 20× and 1 μL of the dilution was used for a PCR template. The design of all primers used the SYBR green system (KAPA SYBR FAST qPCR Kits, KK4600, Kapa Biosystems) and their sequences are listed in SI Appendix, Table S7.

**Mosquito fertility assay**

Three days after blood feeding, female mosquitoes (N=37~42) were anesthetized via ice sedation for 5 minutes before being transferred into *Drosophila* vials containing 3 mL water and 3 X 2 cm filter paper and allowed to lay eggs for 24 hours. The eggs were then counted. Eggs were subsequently hatched and larvae counted three days after egg maturation.

**Mosquito survival rates following exposure to *S. marcescens***

*S. marescens* were cultured from the midguts of *Aedes aegypti* Higgs strain on sheep blood agar plates (BD Multipurpose Culture Medium (Sterile); Nippon Becton Dickinson Company, Ltd., Japan) and identified by VITEK 2 (bioMérieux). *S. mar* were cultured on Luria-Bertani (LB) plates and LB liquid medium was used for amplification. Mosquitoes were fed with antibiotic (10% sucrose solution including 20 units of penicillin and 20 μg of streptomycin per mL on a moistened cotton ball) for three days (Xiao et al., 2017). The mosquitoes were then starved for 16 hours before the bacterial challenge. LB or 50K/mL *S. marescens* were used to feed antibiotic-treated mosquitoes and survival rates were recorded for 12 days. To analyze survival rates we created a Cox Proportional Hazards model in which survival ~ genotype*treatment group in order to enable investigation into interactions between the variables. Survival analyses used data across two biological repeats for each group (total sample sizes=95).

**Immunostaining of *A. aegypti* ovaries and midgut**

The fixing and staining procedures were performed as previously described for *Drosophila* ovaries (Luo et al., 2015). In brief, 5- to 7-day-old Higgs strain or mutant mosquitoes were collected and dissected in 1×PBS to obtain ovaries or midguts. Ovaries or midguts were fixed with 4% paraformaldehyde/PBS for 20 minutes at room temperature and rinsed three times in

PBST (0.1% Triton-X in PBS). Ovaries and midguts were then blocked in 5% NGS (5% normal goat serum in PBST) for at least 30 minutes before incubation with primary antibodies (VASA, 1:500, generated in Yu Cai lab; NICD, C17.9C6, 1:50, Developmental Studies Hybridoma Bank, DSHB and Cleaved-Caspase-3 ,1:500, Cell Signaling Technology, Inc., Massachusetts, USA) diluted in 5% NGS for four hours at room temperature or overnight at 4 °C. The ovaries or midguts were then rinsed and washed with PBST for at least 30 minutes prior to incubation with secondary antibodies (Cy3-conjugated goat against mouse secondary, 1:400, Jackson Immuno Research Laboratories, Inc; Alexa Fluor 555 Phalloidin, 1:400, ThermoFisher Scientific, California, USA) diluted in PBST for 2–3 hours at room temperature.

After incubation with secondary antibodies, ovaries or midguts were rinsed and washed three times with PBST. Samples were incubated with Hoechst 33258 (Invitrogen, California, USA) for 30 minutes before being stored in Vectashield antifade mounting medium (Vector Laboratories, California, USA). Samples were mounted on slides for analysis and images were captured with a Leica SP8 upright confocal microscope. Confocal images were processed using Adobe Photoshop CS6 and Adobe Illustrator CS6 (Adobe Systems).

**dsRNA synthesis and injection**

dsRNA was synthesized following the MEGAscript Kit protocol. The DNA template used an T7 RNA polymerase promoter site upstream of the sequence to be transcribed (Table S8). DNA from the whole mosquito was used as a PCR template. Four reactions were utilized per gene, with each reaction having a 200 ng PCR-product template for transcription reaction assembly. dsRNA was synthesized and incubated at 37 °C for 14 hours. Phenol: chloroform extraction and isopropanol precipitation was used to purify the dsRNA, which was stored frozen at –20 °C.

Control and *GCTL-3*$^{-/-}$ female mosquitoes were exposed to the 1.5 μg dsRNA via thoracic injection (Drummond Nanoject II Auto-Nanoliter Injector) for three days. Females were allowed to lay eggs onto wet filter paper. Eggs were then counted and hatched, and the number of larvae that emerged over the next three days was also counted.

**Follicle analysis**

Ovaries were transferred to glass slides containing 20 μL of Vectashield antifade mounting medium. Individual ovarioles were separated using tungsten needles under a dissection microscope. A single section image or a Z-stack of images were acquired using a Leica SP8 upright confocal. A control mosquito follicle was defined as a follicle containing seven large polyploid nurse cells (NCs) and one meiotically arrested oocyte (OC), as mosquito germline stem cells/progenitors undergo three rounds of synchronized division with incomplete cytokinesis. Germ cell division will generate a follicle with 15 NCs and 1 OC, while a reduced

germ cell division will produce a follicle with 3 NCs and 1 OC. An "encapsulation defect" was defined as two consecutive follicles both containing NCs: OC ratios other than 3:1, 7:1, and 15:1. A "defect in oocyte specification" was defined as a follicle containing 4, 8, or 16 NCs without an OC, while its neighboring follicle was a control follicle. An "agametic germarium" was defined as a germarium without any VASA-positive germ cells.

**Statistical analysis** (Zhang et al., 2017)

Mosquitoes were randomly assigned into different groups. A significance level of $p<0.05$ was used throughout (*$p<0.05$; **$p<0.01$; ***$p<0.001$; ****$p<0.0001$). All data sets were first tested for normality using Shapiro-Wilks tests; normally distributed data sets were assessed using Two-way ANOVA whilst non-normally distributed data sets were assessed using non-parametric Mann–Whitney tests. A Cox Proportional Hazard model was used to compare the survival distributions of multiple populations. ANOVA tests were used for comparisons of egg counts and larval hatches for the *Attacin* and *Gambicin* knock-down experiments. Three biological replicates were conducted for all experiments. Statistical analysis was conducted using the GraphPad Prism 6 statistical software.

**<u>Supplemental References</u>**

Anderson, M.A., Gross, T.L., Myles, K.M., and Adelman, Z.N. (2010). Validation of novel promoter sequences derived from two endogenous ubiquitin genes in transgenic Aedes aegypti. Insect Mol Biol *19*, 441-449.

Das, S., Garver, L., and Dimopoulos, G. (2007). Protocol for mosquito rearing (A. gambiae). J Vis Exp, 221.

Hall, A.B., Basu, S., Jiang, X., Qi, Y., Timoshevskiy, V.A., Biedler, J.K., Sharakhova, M.V., Elahi, R., Anderson, M.A., Chen, X.G., *et al.* (2015). SEX DETERMINATION. A male-determining factor in the mosquito Aedes aegypti. Science *348*, 1268-1270.

Iseli, C., Ambrosini, G., Bucher, P., and Jongeneel, C.V. (2007). Indexing strategies for rapid searches of short words in genome sequences. PLoS One *2*, e579.

Kent, W.J., Sugnet, C.W., Furey, T.S., Roskin, K.M., Pringle, T.H., Zahler, A.M., and Haussler, D. (2002). The human genome browser at UCSC. Genome Res *12*, 996-1006.

Kistler, K.E., Vosshall, L.B., and Matthews, B.J. (2015). Genome engineering with CRISPR-Cas9 in the mosquito Aedes aegypti. Cell Rep *11*, 51-60.

Kondo, S., and Ueda, R. (2013). Highly improved gene targeting by germline-specific Cas9 expression in Drosophila. Genetics *195*, 715-721.

Kyrou, K., Hammond, A.M., Galizi, R., Kranjc, N., Burt, A., Beaghton, A.K., Nolan, T., and Crisanti, A. (2018). A CRISPR-Cas9 gene drive targeting doublesex causes complete population suppression in caged Anopheles gambiae mosquitoes. Nat Biotechnol *36*, 1062-1066.

Lobo, N.F., Clayton, J.R., Fraser, M.J., Kafatos, F.C., and Collins, F.H. (2006). High efficiency

germ-line transformation of mosquitoes. Nat Protoc *1*, 1312-1317.
Luo, L., Wang, H., Fan, C., Liu, S., and Cai, Y. (2015). Wnt ligands regulate Tkv expression to constrain Dpp activity in the Drosophila ovarian stem cell niche. J Cell Biol *209*, 595-608.
Mazaika, E., and Homsy, J. (2014). Digital Droplet PCR: CNV Analysis and Other Applications. Curr Protoc Hum Genet *82*, 7 24 21-13.
Nijole Jasinskiene, J.J., Anthony A. James (2007). Microinjection of A. aegypti Embryos to Obtain Transgenic Mosquitoes. In J Vis Exp.
Xiao, X., Yang, L., Pang, X., Zhang, R., Zhu, Y., Wang, P., Gao, G., and Cheng, G. (2017). A Mesh-Duox pathway regulates homeostasis in the insect gut. Nat Microbiol *2*, 17020.
Zhang, R., Zhu, Y., Pang, X., Xiao, X., Zhang, R., and Cheng, G. (2017). Regulation of Antimicrobial Peptides in Aedes aegypti Aag2 Cells. Front Cell Infect Microbiol *7*, 22.
